# Multimodal Deep Learning for Pulmonary Nodule Detection on Chest Radiography in High‐Risk Adults, With Secondary Validation for All‐Cause and Cause‐Specific Mortality Prediction: A Multicenter Cohort Study

**DOI:** 10.1002/mco2.70730

**Published:** 2026-04-08

**Authors:** Junxian Li, Yuchen Xing, Ximin Gao, Ya Liu, Liwen Zhang, Yubei Huang, Pengyu Zhang, Zhaoxiang Ye, Meng Wang, Fengju Song

**Affiliations:** ^1^ Department of Blood Transfusion Key Laboratory of Cancer Prevention and Therapy in Tianjin National Clinical Research Center for Cancer Tianjin's Clinical Research Center for Cancer Tianjin Medical University Cancer Institute and Hospital Tianjin Medical University Tianjin China; ^2^ Department of Cancer Epidemiology and Biostatistics, Tianjin Key Laboratory of Molecular Cancer Epidemiology, Clinical Research Center for Cancer, Key Laboratory of Cancer Prevention and Therapy, Tianjin's Clinical Research Center For Cancer Tianjin Medical University Cancer Institute and Hospital, Tianjin Medical University Tianjin China; ^3^ Public Health Science and Engineering College Tianjin University of Traditional Chinese Medicine Tianjin China; ^4^ Department of Epidemiology and Statistics, School of Public Health Hebei Medical University, Hebei Key Laboratory of Environment and Human Health Shijiazhuang China; ^5^ Department of Radiology National Clinical Research Centre for Cancer Key Laboratory of Cancer Prevention and Therapy Tianjin's Clinical Research Center for Cancer Tianjin Medical University Cancer Institute and Hospital, Tianjin Medical University Tianjin China; ^6^ Department of Lung Cancer, Key Laboratory of Cancer Prevention and Therapy National Clinical Research Center for Cancer Tianjin's Clinical Research Center for Cancer Tianjin Medical University Cancer Institute and Hospital, Tianjin Medical University Tianjin China

**Keywords:** chest X‐ray, deep learning, lung cancer screening, mortality prediction, pulmonary nodule

## Abstract

Chest radiographs (CXRs) may encode prognostic signals beyond pulmonary nodule detection. We developed LungProNet, a multimodal deep‐learning (DL) model that fuses CXR features with four epidemiologic variables (age, sex, smoking history, and family history) for pulmonary nodule detection as the primary task, with secondary validation for all‐cause and cause‐specific mortality prediction. LungProNet was trained and internally validated on Tianjin Lung Cancer Imaging Dataset (TLCID) (70/30; *n* = 2852/1227) and externally validated on ChestDR (*n* = 4848), with stratified analyses across epidemiologic strata. Discrimination was quantified by area under the curve (AUC) (95% confidence intervals), with accuracy, sensitivity, and specificity reported, and results were benchmarked against contemporary machine learning/DL baselines. The pretrained multimodal encoder was transferred without fine‐tuning to the Prostate, Lung, Colorectal and Ovarian Cancer Screening Trial (PLCO) (*n* = 24,697); its fused embeddings were used as covariates in Cox proportional‐hazards models, and time‐dependent AUCs were evaluated at 1–12 years. For nodule detection, AUCs were 0.979 (0.975–0.982) in TLCID and 0.849 (0.835–0.862) in ChestDR; the TLCID stratified model reached 0.990 (0.984–0.994). In PLCO, AUCs were 0.925 (0.892–0.952) for all‐cause mortality and 0.939–0.985 for cardiac‐, lung cancer‐, and Chronic Obstructive Pulmonary Disease (COPD)‐cause mortality, with robust subgroup performance. These results support CXR‐based nodule flagging within screening workflows and suggest secondary opportunistic risk stratification potential.

## Graphical abstract

The LungProNet deep learning model integrates chest X‐ray imaging and epidemiological data to accurately detect pulmonary nodules and predict all‐cause and cause‐specific mortality in high‐risk adults, demonstrating superior performance compared to traditional models.

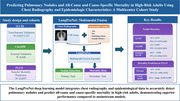

## 1 Introduction

Lung cancer continues to account for the greatest number of cancer deaths globally, and China bears an especially heavy share of this burden [1]. One major driver is insufficient population‐level early‐detection coverage, which is reflected in a lower proportion of Stage I diagnoses in China compared with high‐income settings (17.3% vs. 25.3%) [2, 3]. Accordingly, accurate recognition of pulmonary nodules—often the earliest imaging clue of malignant transformation—is essential for prompt clinical action. Chest X‐ray/chest radiograph (CXR) is still the most widely available and inexpensive first‐line examination; however, it can fail to reveal a substantial fraction of cancers [4], and its interpretation varies considerably across readers [5, 6]. Despite these drawbacks, CXR is performed at very high volume in routine care (e.g., 1039 exams per 1,000 US Medicare Part B beneficiaries in 2013) [7]. In many low‐ and middle‐income contexts, CXR may be the only feasible imaging option for initial triage, so improving its screening yield has direct public‐health relevance.

Low‐dose computed tomography (LDCT) reduces lung‐cancer mortality in high‐risk groups, yet scaling it widely is limited by equipment costs, workflow capacity, and uneven scanner access in resource‐limited areas [8, 9]. In addition, indeterminate LDCT findings can trigger frequent follow‐up testing and heighten patient anxiety, adding workload and financial pressure to healthcare systems. Therefore, scalable and low‐cost approaches that strengthen the clinical value of routine CXR are still needed, particularly for community screening and primary‐care environments.

Beyond visible nodules, CXRs may encode prognostic information that is easy to overlook; for example, subtle markers such as vascular calcification [10] or cardiomegaly [11] on films read as “normal” have been linked to long‐term outcomes, but feedback loops to reinforce these signals are uncommon. This “latent‐signal” view implies that algorithmic analysis could capture clinically meaningful patterns that are not reliably recognized during routine reading. Crucially, any such benefit must hold across different scanners, acquisition protocols, and populations—conditions under which many prior CXR‐artificial intelligence (AI) reports remain under‐validated.

Deep learning (DL) has reached expert‐level sensitivity for very small nodules on CT (down to ∼3 mm) [8]. Yet CT‐centric progress does not readily translate to many underserved regions (including parts of rural China) where CT access is limited [9], leaving CXR as the practical imaging backbone. CXR‐focused DL models remain challenging partly because projection imaging introduces overlapping anatomy (ribs, vessels) that obscures weak signals [12], and partly because many pipelines omit basic risk context that can materially improve discrimination [13, 14]. Moreover, heterogeneous labeling standards and variable image quality across datasets can degrade reproducibility and impede clinical translation, underscoring the need for careful external validation and harmonized evaluation.

**FIGURE 1:**
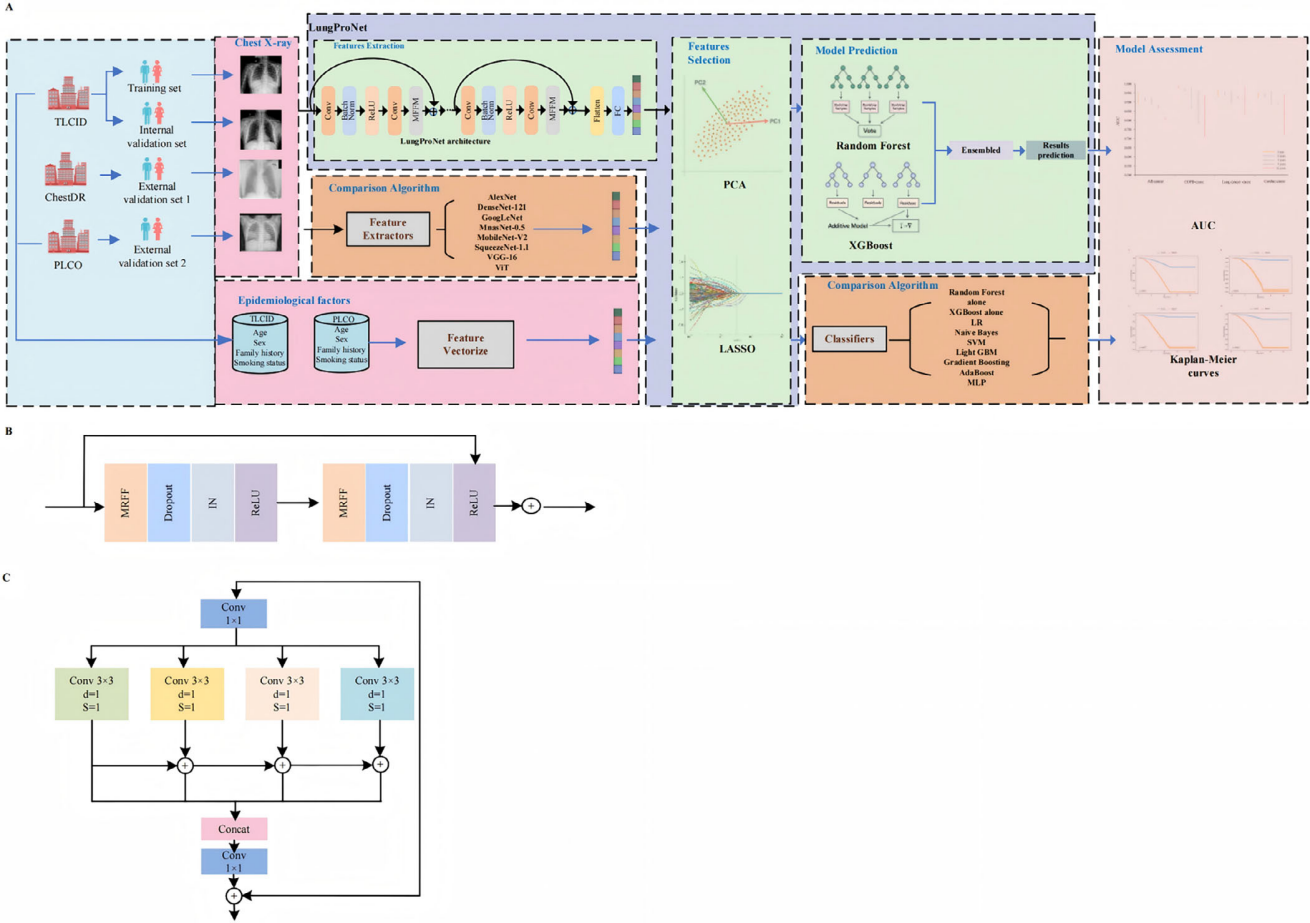
Overview of the workflow of this study. **(A)** The flowchart shows the process of establishing predictive models. We constructed a deep learning model named LungProNet using 70% of the samples from the Tianjin Lung Cancer Imaging Dataset (TLCID) cohort as the training set. We then tested its performance in predicting lung nodules using the remaining 30% of the TLCID cohort as the internal validation set and an external validation set drawn from the ChestDR cohort. Next, we established stratified models by integrating epidemiological information within the TLCID cohort and evaluated its performance in predicting all‐cause mortality and cause‐specific mortality using the Prostate, Lung, Colorectal and Ovarian Cancer Screening Trial (PLCO) cohort. Chest X‐rays (CXRs) served as inputs, from which deep learning features were first extracted and standardized by the multiscale feature fusion module (MFFM) algorithm. Subsequently, principal component analysis (PCA) and the Least Absolute Shrinkage and Selection Operator (LASSO) were applied in parallel to select important deep learning features. Two classifiers—random forest (RF) and extreme gradient boosting (XGBoost)—were trained using the selected features. Finally, an ensemble classifier that averaged the probabilities produced by the trained RF and XGBoost models was used to identify lung nodules. The constructed LungProNet model was compared with various mainstream deep learning and machine learning algorithms in terms of feature extraction and classifier performance, and its performance was evaluated using AUC, accuracy, sensitivity, and specificity. **(B)** Illustration of the MFFM block structure. This panel depicts the two multireceptive field fusion (MRFF) blocks that compose the MFFM module. Each MRFF block uses a 1 × 1 convolution layer to reduce the number of input channels, followed by dilated convolutional layers to extract spatial features at various receptive fields. Dropout and instance normalization (IN) are applied to regularize the feature learning process, while the Rectified Linear Unit (ReLU) activation function is used for non‐linearity. The residual learning technique ensures that the output of the MRFF block is added back to the input, mitigating potential network degradation. **(C)** Illustration of the fusion and convolution operation. This panel demonstrates the fusion of multiple spatial features extracted by several convolutional layers. The diagram highlights the use of three 3 × 3 convolution layers with dilation factors (d = 1) to capture different receptive fields. These features are then fused by concatenation. A final 1 × 1 convolution layer is applied to reduce the channel dimensions after fusion. The outputs of these convolutions are added together, employing residual learning to enhance the overall feature extraction and prevent performance degradation. Here, d denotes the dilation factor and S denotes the convolution stride.

Epidemiologic variables shape both baseline risk and the meaning of imaging cues; for instance, similar‐appearing textures may imply different malignancy probabilities depending on smoking exposure [15, 16, 17], but such interactions are rarely modeled explicitly in CXR‐DL systems. In practice, clinicians synthesize the film with readily available context (age, smoking history, family history), whereas image‐only AI may overlook clinically important cross‐modal effects. Here, we propose and externally validate a multimodal DL framework—Lung Prognosis Network (LungProNet)—that combines CXR‐derived representations with four routinely obtainable variables (age, sex, smoking history, and family history) to enhance pulmonary‐nodule detection on CXR (primary aim), and we further assess its prognostic value for all‐cause and cause‐specific mortality in high‐risk populations (secondary aim) (Figure 1). By prioritizing multimodal fusion and rigorous external testing, we aim to strengthen the real‐world utility of CXR‐based screening where CT‐based programs are difficult to implement.

## 2 Results

### 2.1 Patient Characteristics

As shown in Table 1, among the 4079 patients from the Tianjin Lung Cancer Imaging Dataset (TLCID), 2102 had negative CXR findings, and 1977 had positive findings, with a mean age of 51.55 ± 13.03 years. Compared with patients who had negative CXR results, those with positive results were more likely to be older (58.03 ± 10.67 years), female (50.3%), have a family history of lung cancer (8.7%), and to have smoked more than 30 pack‐years (31.0%). In the Prostate, Lung, Colorectal and Ovarian (PLCO) Cancer Screening Trial cohort, 24,697 participants were included, 21,015 of whom survived during the follow‐up period and 3682 died. The mean age at baseline was 62.59 ± 5.41 years. Compared with survivors, individuals who died were more likely to be older (65.44 ± 5.37 years), male (66.9%), have a family history of lung cancer (11.8%), and to have smoked more than 30 pack‐years (58.1%). Finally, in the ChestDR cohort, 665 of 4848 individuals were CXR‐positive (13.7%), whereas 4183 were CXR‐negative (86.3%).

**TABLE 1:** Baseline characteristics of the study population.

	TLCID^a^				PLCO^b^			
Variable	Overall (*N* = 4079)	Negative (*N* = 2102)	Positive (*N* = 1977)	*p*	Overall (*N* = 24,697)	Negative (*N* = 21,015)	Positive (*N* = 3682)	*p*
Age (mean (SD))	51.55 ± 13.03	45.45 ± 12.06	58.03 ± 10.67	**< 0.001**	62.59 ± 5.41	62.09 ± 5.26	65.44 ± 5.37	**< 0.001**
Sex (%)								
Female	1493 (36.6)	499 (23.7)	994 (50.3)	**< 0.001**	11,938 (48.3)	10,721 (51.0)	1217 (33.1)	**< 0.001**
Male	2586 (63.4)	1603 (76.3)	983 (49.7)		12,759 (51.7)	10,294 (49.0)	2465 (66.9)	
Family history of lung cancer (%)								
No	3780 (92.7)	1975 (94.0)	1805 (91.3)	**0.001**	21,916 (88.7)	18,669 (88.8)	3247 (88.2)	0.261
Yes	299 (7.3)	127 (6.0)	172 (8.7)		2781 (11.3)	2346 (11.2)	435 (11.8)	
Smoking history (%)								
Never	3104 (76.1)	1891 (90.0)	1213 (61.4)	**< 0.001**	10,724 (43.4)	9698 (46.1)	1026 (27.9)	**< 0.001**
< 30 pack‐years	235 (5.8)	83 (3.9)	152 (7.7)		4813 (19.5)	4297 (20.4)	516 (14.0)	
≥ 30 pack‐years	740 (18.1)	128 (6.1)	612 (31.0)		9160 (37.1)	7020 (33.4)	2140 (58.1)	

Abbreviations: PLCO, The Prostate, Lung, Colorectal and Ovarian Cancer Screening Trial; SD, standard deviation; TLCID, Tianjin Lung Cancer Imaging Dataset.

^a^ Patients with pulmonary nodules are defined as positive, while patients without pulmonary nodules are defined as negative.

^b^ Participants who died during the follow‐up within 12 years are defined as positive, while participants who survived the 12‐year follow‐up period are defined as negative.

Bold values indicate statistical significance *p* < 0.05.

### 2.2 Performance Comparison of LungProNet Models for Identifying Pulmonary Nodules

In the task of identifying pulmonary nodules, our model demonstrated superior performance. In the training set, the model achieved an area under the curve (AUC) of 0.990 (95% confidence intervals [CI]: 0.989–0.991), with an overall accuracy of 0.931. Additionally, the sensitivity and specificity were 0.987 and 0.928, respectively. In the TLCID set, our DL model accurately identified CXRs containing pulmonary nodules and achieved an AUC of 0.979 (95% CI: 0.975–0.982), an accuracy of 0.928, a sensitivity of 0.922, and a specificity of 0.928. Performance was comparable in the ChestDR set, where the model achieved an AUC of 0.849 (95% CI: 0.835–0.862) (Table 2). We also developed several other DL and machine learning (ML) models for comparison, and the results are summarized in Tables S1 and S2. Among all the models, our model achieved the highest AUC, demonstrating its strong diagnostic performance.

**TABLE 2:** Performance of the LungProNet model for detecting pulmonary nodules.

Cohort	AUC (95% CI)	Accuracy	Sensitivity	Specificity
Training set	0.990 (0.989–0.991)	0.931	0.987	0.928
Internal validation set				
Entire	0.979 (0.975–0.982)	0.928	0.922	0.928
Strata				
Age				
< 55 years	0.960 (0.951–0.968)	0.900	0.847	0.950
55–74 years	0.968 (0.964–0.971)	0.976	0.682	0.990
> 74 years	0.990 (0.984–0.994)	0.971	0.825	0.992
Sex				
Female	0.975 (0.971–0.978)	0.974	0.735	0.990
Male	0.964 (0.960–0.968)	0.975	0.685	0.992
Family history of lung cancer				
No	0.969 (0.966–0.972)	0.975	0.725	0.991
Yes	0.974 (0.966–0.980)	0.969	0.645	0.990
Smoke pack‐years				
Never	0.972 (0.969–0.975)	0.972	0.785	0.985
≤ 30 pack‐years	0.971 (0.963–0.978)	0.978	0.655	0.995
> 30 pack‐years	0.963 (0.957–0.969)	0.975	0.725	0.991
External validation set 1	0.849 (0.835–0.862)	0.725	0.940	0.625

Abbreviations: AUC, area under the curve; CI, confidence intervals.

### 2.3 Performance Comparison of LungProNet Models in Stratified Analysis

To demonstrate the ability of LungProNet to identify pulmonary nodules across diverse populations, we built stratified variants by integrating key epidemiological variables. Following National Lung Screening Trial (NLST) criteria for high‐risk lung‐cancer screening, we categorized patients into three age groups (< 55, 55–74, > 74 years); two sex groups (male and female); two groups for family history of lung cancer (yes, no); and three groups for cumulative smoking exposure (never, ≤ 30, > 30 pack‐years). Within each stratum, we evaluated the model's performance using the TLCID dataset (Table 2). In the stratified analysis, our model yielded the highest AUC of 0.990 (95% CI: 0.984–0.994) among individuals aged > 74 years, whereas the greatest accuracy (0.978) was obtained in participants with ≤ 30 pack‐years of smoking. Across all strata, AUC, accuracy, and specificity remained consistently high, underscoring the robustness of our approach.

### 2.4 Integration of Epidemiological Factors Into the LungProNet Model for Predicting all‐Cause and Cause‐Specific Mortality

**FIGURE 2:**
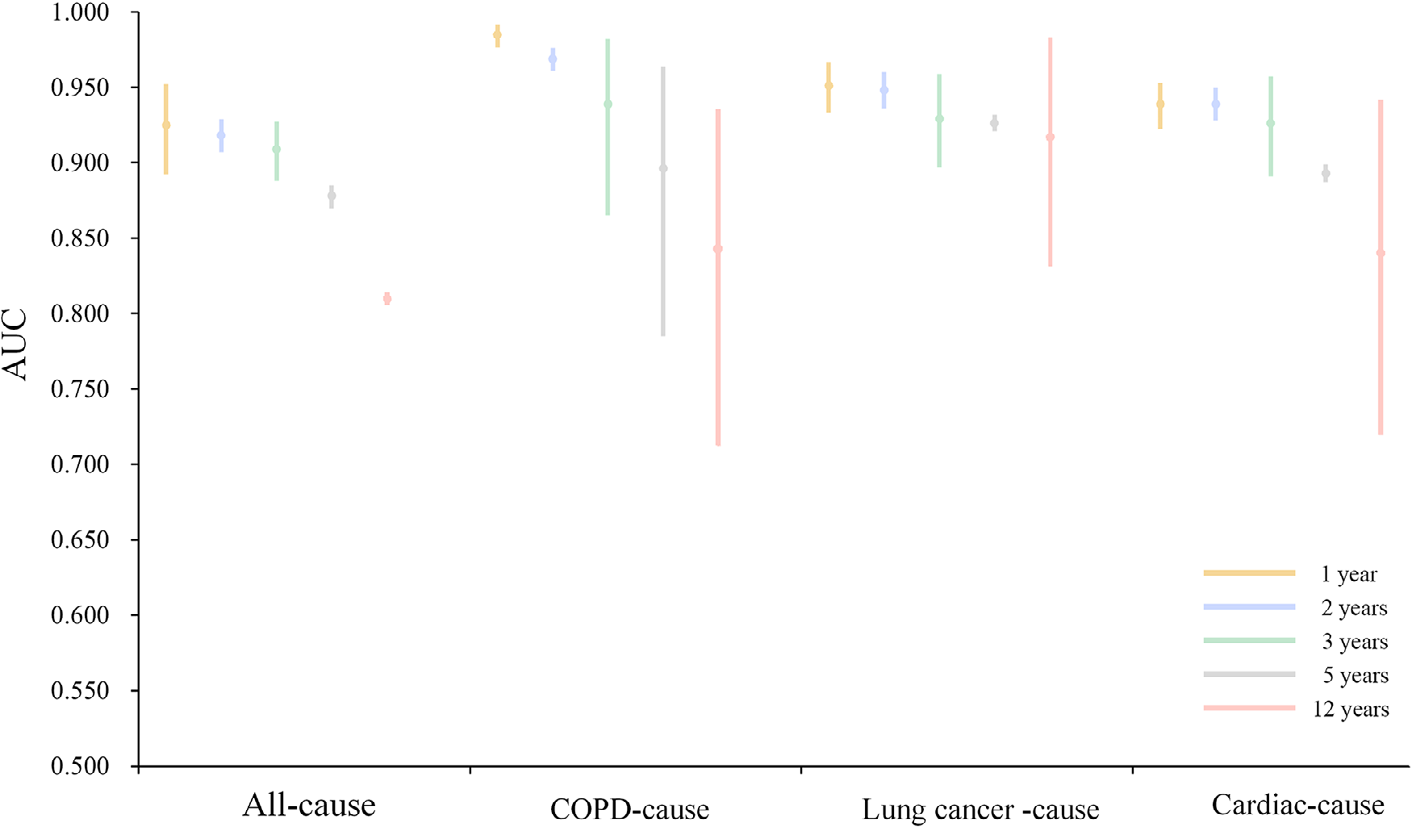
Comparison of the area under the receiver‐operating‐characteristic curves of LungProNet model in predicting all‐cause, chronic obstructive pulmonary disease, lung cancer, and cardiac‐cause mortality.

As secondary validation in the PLCO cohort, to maximize the effectiveness of our model in resource‐limited settings, we integrated CXR images with four key epidemiological variables—age, sex, family history of lung cancer, and smoking history—to develop a multimodal model capable of predicting short‐ and long‐term mortality risks. The results are presented in Table 3 and Figure 2.

**TABLE 3:** Performance of LungProNet model for predicting all‐cause mortality and cause‐specific mortality.

Time horizon	All‐cause			COPD‐cause			Lung cancer‐cause			Cardiac‐cause		
	Accuracy	Sensitivity	Specificity	Accuracy	Sensitivity	Specificity	Accuracy	Sensitivity	Specificity	Accuracy	Sensitivity	Specificity
1 year	0.999	0.748	0.992	0.996	0.793	0.988	0.952	0.752	0.953	0.948	0.784	0.962
2 years	0.990	0.712	0.991	0.986	0.708	0.985	0.939	0.703	0.947	0.935	0.758	0.951
3 years	0.996	0.684	0.993	0.982	0.751	0.974	0.927	0.782	0.935	0.952	0.763	0.937
5 years	0.970	0.658	0.971	0.971	0.749	0.965	0.910	0.628	0.928	0.847	0.802	0.861
12 years	0.861	0.635	0.865	0.948	0.603	0.953	0.892	0.715	0.910	0.874	0.682	0.875

Abbreviations: AUC, area under the curve; COPD, chronic obstructive pulmonary disease.

When applied to all‐cause mortality prediction, the model demonstrated remarkable performance. Specifically, it achieved AUCs of 0.925 (95% CI: 0.892–0.952), 0.918 (95% CI: 0.907–0.929), 0.909 (95% CI: 0.888–0.927), 0.878 (95% CI: 0.870–0.885), and 0.810 (95% CI: 0.806–0.814), with corresponding accuracies of 0.999, 0.990, 0.996, 0.970, and 0.861 for prediction windows of 1, 2, 3, 5, and 12 years, respectively. For predicting death from Chronic Obstructive Pulmonary Disease (COPD), the model achieved AUCs of 0.985 (95% CI: 0.976–0.992), 0.969 (95% CI: 0.961–0.976), 0.939 (95% CI: 0.865–0.982), 0.896 (95% CI: 0.785–0.964), and 0.843 (95% CI: 0.712–0.935), with accuracies of 0.996, 0.986, 0.982, 0.971, and 0.948 across the same time horizons. For predicting death from lung cancer, AUCs were 0.951 (95% CI: 0.933–0.967), 0.948 (95% CI: 0.936–0.960), 0.929 (95% CI: 0.897–0.959), 0.926 (95% CI: 0.921–0.932), and 0.917 (95% CI: 0.832–0.983), with accuracies of 0.952, 0.939, 0.927, 0.910, and 0.892, respectively. For predicting death from cardiac causes, AUCs were 0.939 (95% CI: 0.922–0.953), 0.939 (95% CI: 0.928–0.950), 0.926 (95% CI: 0.891–0.957), 0.893 (95% CI: 0.887–0.899), and 0.840 (95% CI: 0.719–0.940), with accuracies of 0.948, 0.935, 0.952, 0.847, and 0.874, respectively.

**FIGURE 3:**
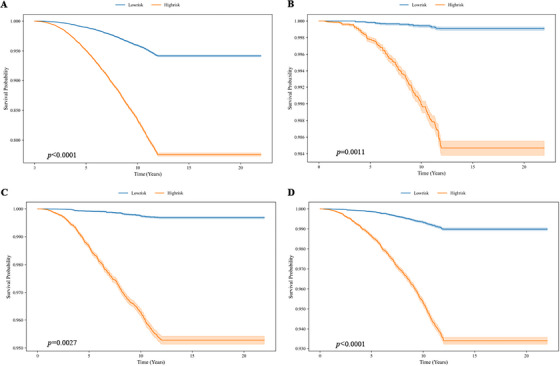
Kaplan–Meier curves of LungProNet model in predicting (A) all‐cause, (B) chronic obstructive pulmonary disease, (C) lung cancer, and (D) cardiac causes mortality.

Tables S3–S6 summarize the DL comparisons, whereas Tables S7–S10 summarize the ML comparisons. Across all tasks, LungProNet consistently achieved the highest AUCs, underscoring its superior performance. Figure 3 illustrates the relationship between LungProNet predictions and observed mortality after incorporating epidemiological factors. Across all causes of death, survival was significantly lower in the high‐risk group than in the low‐risk group (*p* ≤ 0.0027).

## 3 Discussion

This study developed LungProNet, a DL model that extracts imaging features from TLCID CXRs. These image‐derived features were combined with four epidemiological variables—age, sex, smoking history, and family history of lung cancer—to enable stratified analyses and predict pulmonary nodule occurrence. Several ML algorithms, notably random forest (RF) and extreme gradient boosting (XGBoost), were applied to rank and select the most informative DL features, thereby optimizing model performance. The resulting classifiers were ensembled voting, yielding final probabilities. Internal validation using TLCID data yielded an AUC of 0.979 (95 % CI 0.975–0.982), while external validation with ChestDR data produced an AUC of 0.849 (95 % CI 0.835–0.862). In stratified analyses, the integrated DL–epidemiological model achieved a peak AUC of 0.990 (95 % CI 0.984–0.994) and remained stable across subgroups. When applied to the PLCO cohort, the model not only predicted all‐cause mortality but also demonstrated potential for cause‐specific mortality, particularly lung‐cancer‐related death, with AUCs of 0.951, 0.948, 0.929, 0.926, and 0.917 for the 1‐, 2‐, 3‐, 5‐, and 12‐year horizons, respectively (corresponding 95% CIs: 0.933–0.967; 0.936–0.960; 0.897–0.959; 0.921–0.932; 0.832–0.983). These findings suggest that integrating epidemiological characteristics with chest‐radiograph features provides precise support for early pulmonary‐nodule screening and prognostic assessment. Consistent with the study aim, LungProNet is primarily positioned for CXR‐based pulmonary nodule detection in screening settings, with mortality prediction reported as secondary validation of prognostic utility.

Given the global burden of lung cancer, chest radiography remains one of the first‐line modalities for screening and follow‐up. However, as the number of cases continues to rise, radiologists are under increasing strain from the growing workload. AI systems can not only ease this workload and enhance diagnostic accuracy by flagging subtle lesions that human readers may miss, they can also provide prognostic insights. For example, De Margerie‐Mellon et al. reported promising AI performance across several chest‐radiograph applications involving lung nodules and lung cancer [18]. Similarly, Gandhi et al. demonstrated that AI could predict treatment responses, supporting clinical decision‐making and helping clinicians choose the optimal treatment plan [19].

In daily practice, integrating AI to assist physicians in nodule detection on CXRs has therefore become essential. The DL model developed by Nam et al. outperformed radiologists, achieving a highest Jackknife Alternative Free‐response Receiver Operating Characteristic (JAFROC) score of 0.924 [20]. Steven et al. validated a DL model using data from four institutions, which helped radiologists detect pulmonary nodules and increased their mean AUC to 0.841 [21]. Sim et al. reported that the average radiologist sensitivity rose to 0.703 when assisted by AI algorithm [22]. Nevertheless, overall performance remains sub‐optimal. By contrast, our CXR‐only model achieved AUCs of 0.979 on the TLCID set and 0.849 on the ChestDR set.

Because the NLST already stratifies high‐risk patients according to epidemiological variables, we incorporated age, sex, smoking history, and family history of lung cancer into our network and conducted subgroup analyses based on NLST strata. The results demonstrated that our model maintained strong performance across every stratum, with AUCs between 0.960 and 0.990. In addition, the model's accuracy and specificity were consistently high. The model not only delivers accurate overall predictions but also demonstrates high reliability when identifying negative cases. This ability to confidently rule out disease can help physicians avoid unnecessary interventions, ease financial burdens, and reduce patient anxiety, underscoring the model's clinical value.

Earlier efforts to extract prognostic information from CXRs have been promising but remain limited in scope. Lu et al. showed that an Inception‐v4 model could predict overall mortality from CXRs [23]. But the performance of their model was reported only for short‐term outcomes and without adjustment for established epidemiologic risk factors. Weiss et al. subsequently refined this idea, training a network that estimated mortality risk in several pulmonary diseases [24]. However, each disease of the model was modeled in isolation and external validation, although attempted, relied on relatively small cohorts. Their follow‐up study extended the approach to cardiovascular events, but again treated cardiac deaths as a stand‐alone endpoint and ignored competing respiratory causes [25]. Our work addresses these gaps on three fronts. First, we integrate four key epidemiologic variables—age, sex, smoking history and family history of lung cancer—directly into the network, allowing image signals to be interpreted in the context of patient‐level risk. Second, the multimodal architecture produces simultaneous predictions for all‐cause, COPD‐related, lung‐cancer‐specific and cardiac‐cause mortality. Third, the model delivers superior discrimination, with AUCs of 0.951, 0.948, 0.929, 0.926, and 0.917 for the 1‐, 2‐, 3‐, 5‐, and 12‐year horizons, respectively—substantially higher than the figures reported in earlier studies—while maintaining robust performance across epidemiologic strata.

To our knowledge, this study is the first to develop an imaging model based on CXRs from TLCID for the detection of pulmonary nodules, integrating DL features extracted using LungProNet together with four epidemiological factors—age, sex, smoking history, and family history of lung cancer—for stratified analysis. Incorporating these epidemiological variables into the DL model enhances its predictive capability, enabling simultaneous prediction of all‐cause and cause‐specific mortality in the PLCO dataset. This multimodal approach not only enhances diagnostic accuracy but also offers a more comprehensive assessment of patient risk profiles. Furthermore, survival analysis shows that our model maintains robust predictive performance regardless of cause of death. In this study, we employed Least Absolute Shrinkage and Selection Operator (LASSO) for DL feature selection and built predictive models using RF and XGBoost algorithms. Averaging predicted probabilities through a voting mechanism increased the robustness and generalizability of the model. The validation strategy, consisting of internal validation using 30% of patients from TLCID and external validation on the ChestDR dataset, underscores the reliability of our findings across different populations. In addition, this study provides an efficient and convenient solution for community‐based lung cancer screening. Traditional screening methods relying on CT scans are often expensive and entail high radiation doses, making them less feasible for large‐scale community implementation. In contrast, CXRs are cost‐effective and expose patients to lower radiation levels, and—when paired with our model—enable large‐scale pulmonary‐nodule screening together with accurate short‐ and long‐term predictions of all‐cause, COPD‐, lung‐cancer‐, and cardiac‐related mortality. Overall, this strategy holds significant public‐health value by improving early detection and reducing lung‐cancer mortality.

While this study yields significant results, several limitations remain. First, although we assessed model performance on both an internal (TLCID) and an external (ChestDR) cohort, the relatively small sample size may limit the model's generalizability to other ethnic groups. Second, because the model relies solely on chest‐radiograph data and does not incorporate complementary imaging modalities such as CT, its diagnostic accuracy and clinical applicability may be reduced. Third, although advanced DL techniques were employed, the model's limited interpretability continues to hamper actionable clinical decision‐making. Model use will require trained clinical or research staff to verify inputs and interpret outputs in the clinical context. Future research should recruit larger, more diverse cohorts, integrate multimodal imaging inputs (e.g. combining CXRs with CT), and include interpretable ML frameworks to enhance generalizability and clinical uptake. For future implementation, input data should be checked for completeness and quality before model use.

## 4 Conclusion

This study presents a multimodal model that fuses deep‐learning features extracted from CXRs with epidemiological variables to detect the presence of pulmonary nodules during screening rather than stratify their malignant potential. The framework was internally validated on the TLCID cohort and externally tested on the ChestDR cohort, demonstrating robust performance and good generalizability. In the PLCO cohort, the same pre‐trained model retained strong predictive power for both all‐cause and cause–specific mortality, underscoring its potential clinical impact. By uniting imaging data with key demographic and medical‐history factors, the approach brings routine chest‐radiograph screening closer to intelligent, patient‐centered decision support and offers new methodological guidance for early lung‐cancer detection and prognostic assessment.

## 5 Methods

We conducted a retrospective study to test an AI approach for pulmonary‐nodule recognition on CXRs and for estimating long‐term mortality risk. Use of the hospital imaging/clinical data was reviewed and approved by the Institutional Review Board (IRB) of Tianjin Medical University Cancer Institute and Hospital (TMUCIH). For the PLCO Cancer Screening Trial, written informed consent had been obtained in the parent study; the present secondary analysis was approved by the IRB of the U.S. National Cancer Institute (Bethesda, MD). External validation additionally used CXRs from the public ChestDR database; these data are anonymized and released for research under applicable ethical requirements.

### 5.1 Study Design and Participant Cohorts

We analyzed three independent cohorts obtained from separate data sources. Model development used the TLCID in two stages: (i) learning CXR representations with LungProNet and (ii) combining imaging features with epidemiological variables using a hybrid multimodal design. Generalizability for nodule detection was evaluated on ChestDR, and subgroup analyses were performed to examine performance heterogeneity. The third cohort, derived from the PLCO, was included for secondary validation of the model's ability to predict all‐cause and cause‐specific mortality, providing insights into prognostic utility beyond the primary nodule‐detection task (Figure 1).

TLCID was assembled from 4610 individuals undergoing routine examinations at TMUCIH between January 2012 and June 2019 (Figure S1A). Each participant had CXR and CT performed in the same examination period, and radiologists confirmed diagnoses using the imaging work‐up. After removing 531 cases lacking complete epidemiological variables, 4079 CXRs remained for analysis. Data were split at the patient level into training and internal validation sets (7:3), preventing leakage across splits. The resulting partitions contained 2852 patients for training and 1227 patients for internal validation. TLCID served as the development cohort for representation learning and for building the multimodal model with epidemiological covariates.

For external testing of nodule identification, we used the nodule‐labeled subset of ChestDR (External Validation Set 1, Figure S1B). ChestDR is a multicenter Chinese dataset containing 4848 frontal CXRs annotated across 19 thoracic‐abnormality categories. We extracted the nodule category to quantify performance specifically for pulmonary nodules, while preserving the dataset's heterogeneity in clinical presentations and acquisition sites.

The third cohort came from PLCO, a randomized controlled trial (1993–2001) enrolling 154,901 individuals aged 55–74. Participants were randomized to an intervention arm receiving annual CXR screening for up to 4 years or to a usual‐care control arm. From ∼89,000 CXRs representing ∼25,000 participants, we excluded 303 individuals with missing key variables, resulting in 24,697 participants and 88,562 images for analysis (External Validation Set 2, Figure S1C). Outcomes were ascertained using PLCO follow‐up procedures, including questionnaires and medical‐record confirmation [26]. This design allowed us to examine prognostic value for all‐cause and cause‐specific mortality as an additional test of representation transferability.

In brief, LungProNet was trained on TLCID to learn CXR imaging features for pulmonary nodule detection and to build a multimodal encoder by fusing imaging components with epidemiologic variables. The fully specified pipeline (including resizing/normalization, principal‐component analysis [PCA] and LASSO learned on TLCID, and the gated‐attention fusion) was then externally tested on ChestDR to assess generalizability for nodule detection. Finally, without any additional fine‐tuning, the same frozen multimodal encoder was transferred to PLCO CXRs to generate participant‐level fused embeddings, which were used as covariates in Cox models to evaluate all‐cause and cause‐specific mortality, thereby assessing prognostic utility beyond nodule detection. This secondary mortality validation was not intended to replace guideline‐directed management or LDCT‐based diagnostic pathways, but to evaluate generalizability of the pretrained multimodal representation.

### 5.2 Image Acquisition and Processing

We harmonized preprocessing steps across cohorts to minimize differences attributable to file formats and display settings. In TLCID, CXRs were acquired using a Kodak DR3500 digital radiography system in the posteroanterior (PA) view with a 150‐cm source‐to‐detector distance. Typical exposure settings were 105–110 kVp and 8–12 mAs for PA images, and 120 kVp with 12 mAs for lateral views (Table S11). Acquisition time was 3 s; rare parameter adjustments were allowed when necessary, while maintaining Digital Imaging and Communications in Medicine (DICOM)‐Version 3.0 compliance. Post‐processing (e.g., local magnification, grayscale tuning, and spatial‐frequency enhancement) was applied to improve visibility of subtle findings. Window width/level (WW/WL) settings were standardized to support consistent downstream analysis. Quality checks compared a random subset of converted portable network graphics (PNGs) with the corresponding original DICOM images to confirm fidelity.

PLCO images were provided as Tagged Image Format files (TIFF) with protected health information (PHI) redactions; we used ImageMagick 6.8.9‐9 to convert them to PNG and ensured the shorter image dimension was at least 512 pixels. ChestDR images were already distributed as PNG and therefore did not require format conversion.

### 5.3 Outcomes Assessment

In the TLCID, the ground‐truth label for pulmonary nodule presence was defined by LDCT scans (reference standard). Specifically, an index CXR was labeled as positive if the paired LDCT performed in temporal proximity to that CXR reported ≥1 pulmonary nodule, and labeled as negative if LDCT reported no nodule. This definition was applied regardless of whether the nodule was clearly visible on the CXR. Two radiologists (>5 years' experience each) independently reviewed the CXRs. Representative examples by nodule size are shown in Figure S2 (<5 mm, 5–10 mm, and >10 mm; Figure S2A–C). Senior radiologists audited reports and adjudicated disagreements through consensus discussion. When surgery was performed, pathology provided confirmation; otherwise, LDCT findings together with expert radiologist interpretation were used to establish nodule status. Radiologist CXR reads were used for quality control and report adjudication, but LDCT findings served as the label source in TLCID.

In ChestDR, images annotated as containing pulmonary nodules were treated as positive. Annotations were created by residents using standardized rules and then verified by senior radiologists to improve reliability. Resident labeling was performed under supervision to maintain consistency with diagnostic criteria. Senior reviewers re‐checked each case and confirmed or revised labels when necessary.

Unlike TLCID and ChestDR, PLCO provides longitudinal outcomes, enabling an assessment of prognostic relevance. In PLCO, the primary endpoint was all‐cause mortality; follow‐up continued through December 31, 2009 (up to 13 years). For all prognostic analyses, the date of the index (baseline) CXR used for model inference was defined as time zero. Deaths and incident cancers were determined using annual questionnaires, next‐of‐kin reports, and linkage to the National Death Index. Secondary endpoints were cause‐specific mortality from COPD, cardiac causes, and lung cancer, consistent with PLCO definitions [27].

### 5.4 Collection of Epidemiological Characteristics

Four epidemiological variables were extracted from TLCID records. Variables included age (continuous), sex (male/female), family history of lung cancer (yes/no), and smoking exposure (never; < 30 pack‐years; ≥ 30 pack‐years). Pack‐years were computed as packs per day multiplied by years smoked; one pack was defined as 30 cigarettes.

### 5.5 Architecture of LungProNet

We designed LungProNet to detect pulmonary nodules from CXR images (Figure 1A). The backbone follows a residual‐learning design (ResNet‐like), stacking blocks that combine convolution, batch normalization, and Rectified Linear Unit (ReLU) with identity skip connections (Figure 1B,C). Skip connections help preserve informative signals, stabilize gradients, and support deeper training. Before model input, all CXRs are resized to 224 × 224 and intensity‐normalized.

We additionally introduce a multiscale feature fusion module (MFFM) to aggregate information across resolutions and generate a 224 × 224 feature map emphasizing subtle nodule cues. Global average pooling reduces the feature map to a 2048‐dimensional vector capturing the most discriminative image information. To limit overfitting, we apply PCA to the 2,048‐D vector and keep 64 components; PCA parameters [means/standard deviations (SD)] are estimated from the TLCID training set and then reused unchanged for ChestDR and PLCO to maintain cross‐cohort consistency. LASSO is subsequently used to remove residual noise and retain only informative components.

Selected imaging features are combined with epidemiological variables (age, sex, smoking history, family history) using a two‐branch multimodal network. One branch processes the 64‐D image representation, and the other encodes epidemiological inputs via a three‐layer multilayer perceptron (MLP) (32→16→8). A gated‐attention mechanism merges both latent vectors and adaptively re‐weights modalities prior to classification. The fused embedding is then input to RF and XGBoost classifiers in parallel, and their probabilities are combined using soft voting.

### 5.6 Training, Validation, and Benchmark Comparison

An overview of the workflow is provided in Figure 1A. TLCID data were split into 70% training and 30% internal validation using stratified sampling. We trained LungProNet for 50 epochs with stochastic gradient descent (batch size 32; initial learning rate 0.001). External testing was performed on ChestDR using the identical preprocessing and modeling pipeline (resize/normalize→PCA→LASSO→gated fusion→RF+XGBoost ensemble), and ChestDR metrics were used to quantify generalization to unseen populations.

To assess the contribution of the feature extractor, we compared LungProNet against AlexNet, DenseNet‐121, GoogLeNet, MnasNet‐0.5, MobileNet‐V2, SqueezeNet‐1.1, VGG‐16, and a Vision Transformer (ViT). For the classifier, we compared the RF + XGBoost ensemble with logistic regression (LR), naïve Bayes, support vector machine (SVM), standalone RF, standalone XGBoost, light gradient boosting machine (LightGBM), gradient boosting, Adaptive Boosting (AdaBoost), and MLP. Hyperparameters and implementation details are listed in Supporting Information Materials.

Finally, we transferred the pretrained multimodal encoder to PLCO CXRs without further fine‐tuning. The resulting fused embeddings were used as covariates in Cox proportional‐hazards models to evaluate all‐cause and cause‐specific mortality, testing whether the representation captured prognostically relevant information beyond nodule detection.

### 5.7 Model Performance Evaluation Metrics

Model performance was assessed using 10‐fold cross‐validation. For each fold, we computed true positives (TP), true negatives (TN), false positives (FP), and false negatives (FN) counts. Discrimination was summarized primarily using the AUC. We also reported accuracy, sensitivity, and specificity. Metrics were calculated as:

$$\mathrm{Accuracy}=\frac{\mathrm{TP}+\mathrm{TN}}{\mathrm{TP}+\mathrm{TN}+\mathrm{FP}+\mathrm{FN}}$$

$$\mathrm{Sensitivity}=\frac{\mathrm{TP}}{\mathrm{TP}+\mathrm{FN}}$$

$$\mathrm{Specificity}=\frac{\mathrm{TN}}{\mathrm{TN}+\mathrm{FP}}$$

### 5.8 Statistical Analysis

In TLCID, categorical variables were summarized as *n* (%) and compared using the chi‐square test or Fisher's exact test, depending on expected cell counts. Normally distributed continuous variables were presented as the mean ± SD and compared with an independent‐samples *t*‐test. Non‐normal continuous variables were summarized as median (IQR) and compared using the Mann–Whitney *U* test. T for nodule detection, AUC, and 95% CI were estimated using bootstrap resampling (1000 iterations).

In PLCO, baseline characteristics were summarized according to survival status for mortality analyses. Categorical variables were compared using chi‐square or Fisher's exact tests, as appropriate. For continuous variables, we used mean ± SD with *t*‐tests when normality assumptions were met. Otherwise, variables were summarized as median (IQR) and compared using the Mann–Whitney *U* test. Survival analyses evaluated associations between model‐derived risk estimates and mortality endpoints (all‐cause and cause‐specific). Kaplan–Meier curves were plotted, and between‐group differences were tested using the log‐rank statistic. Participants without the event were right‐censored at the last known follow‐up date (or at administrative end of follow‐up, December 31, 2009). Time‐dependent AUCs at 1, 2, 3, 5, and 12 years were calculated from the index CXR (time zero) using censoring‐aware estimation.

All analyses were implemented in Python 3.6; two‐sided *p*‐values < 0.05 were considered statistically significant.

## Author Contributions

Junxian Li, Ya Liu, and Liwen Zhang designed the study. Junxian Li, Ya Liu, Liwen Zhang, and Yubei Huang collected and organized multicenter data. Junxian Li, Yuchen Xing, and Ximin Gao did the statistical analysis and drafted the manuscript. Zhaoxiang Ye and Meng Wang determined the quality of CXRs. Pengyu Zhang and Meng Wang provided pathology diagnostic support. Fengju Song reviewed and revised the manuscript. All authors have read and approved the final manuscript.

## Funding

This study was supported by Chinese National Key Research and Development Project (Grants 2021YFC2500404), The Introduction of talents and doctoral start‐up fund of Tianjin Medical University Cancer Institute and Hospital (Grants B2317), Natural Science Foundation of Hebei Province (Grants H2024206065), and Tianjin Key Medical Discipline Construction Project (Grant No. TJYXZDXK‐3‐003A).

## Ethics Statement

This study was conducted in accordance with Declaration of Helsinki and was approved by the Ethics Committee of Tianjin Medical University Cancer Hospital (Approve number: EK20240287).

## Conflicts of Interest

The authors declare no conflicts of interest.

## Supporting information




**Table S1**. Performance comparison of mainstream DL algorithm applied for feature extractor across validation sets.
**Table S2**. Performance comparison of mainstream ML algorithm applied for classifiers across validation sets.
**Table S3**. Performance comparison of mainstream DL algorithm applied for feature extractor across validation sets for predicting all‐cause mortality.
**Table S4**. Performance comparison of mainstream DL algorithm applied for feature extractor across validation sets for predicting COPD ‐cause mortality.
**Table S5**. Performance comparison of mainstream DL algorithm applied for feature extractor across validation sets for predicting lung cancer ‐cause mortality.
**Table S6**. Performance comparison of mainstream DL algorithm applied for feature extractor across validation sets for predicting cardiac‐cause mortality.
**Table S7**. Performance comparison of mainstream ML algorithm applied for feature extractor across validation sets for predicting all‐cause mortality.
**Table S8**. Performance comparison of mainstream ML algorithm applied for feature extractor across validation sets for predicting COPD‐cause mortality.
**Table S9**. Performance comparison of mainstream ML algorithm applied for feature extractor across validation sets for predicting lung cancer ‐cause mortality.
**Table S10**. Performance comparison of mainstream ML algorithm applied for feature extractor across validation sets for predicting cardiac cause mortality.
**Table S11**. Comprehensive metadata for CXR imaging parameters
**Figure S1**. Flow chart of patient inclusion and exclusion in (A) TLCID, (B) ChestDR and (C) PLCO. CXR: chest X‐ray; TMUCIH, Tianjin Medical University Cancer Institute and Hospital; PLCO: The Prostate, Lung, Colorectal and Ovarian Cancer Screening Trial; TLCID: Tianjin Lung Cancer Imaging Dataset.
**Figure S2**. Representative examples of chest X‐rays with pulmonary nodules of different diameters. **(A)**Pulmonary nodule with a diameter of less than 5 mm.**(B)** Pulmonary nodule with a diameter between 5 mm and 10 mm.**(C)** Pulmonary nodule with a diameter greater than 10 mm.

## Data Availability

Data generated in this study are not publicly available due to compliance restrictions for the protection of patient privacy but are available upon reasonable request from the corresponding author. The PLCO Cancer Screening Trial data are available at https://cdas.cancer.gov/datasets/plco/21/. The ChestDR database is available at: https://springernature.figshare.com/articles/dataset/ChestDR_Thoracic_Diseases_Screening_in_Chest_Radiography/22302775?file=39673366.
