# Supplementary material for: Multimodal Deep Learning for Pulmonary Nodule Detection on Chest Radiography in High‐Risk Adults, With Secondary Validation for All‐Cause and Cause‐Specific Mortality Prediction: A Multicenter Cohort Study
Source: MedComm (2020). 2026 Apr 8;7(4):e70730. doi: 10.1002/mco2.70730 (PMC13062638; doi:10.1002/mco2.70730)
Supplement: Supplementary file 1 — Table S1. Performance comparison of mainstream DL algorithm applied for feature extractor across validation sets. Table S2. Performance comparison of mainstream ML algorithm applied for classifiers across validation sets. Table S3. Performance comparison of mainstream DL algorithm applied for feature extractor across validation sets for predicting all‐cause mortality. Table S4. Performance comparison of mainstream DL algorithm applied for feature extractor across validation sets for predicting COPD ‐cause mortality. Table S5. Performance comparison of mainstream DL algorithm applied for feature extractor across validation sets for predicting lung cancer ‐cause mortality. Table S6. Performance comparison of mainstream DL algorithm applied for feature extractor across validation sets for predicting cardiac‐cause mortality. Table S7. Performance comparison of mainstream ML algorithm applied for feature extractor across validation sets for predicting all‐cause mortality. Table S8. Performance comparison of mainstream ML algorithm applied for feature extractor across validation sets for predicting COPD‐cause mortality. Table S9. Performance comparison of mainstream ML algorithm applied for feature extractor across validation sets for predicting lung cancer ‐cause mortality. Table S10. Performance comparison of mainstream ML algorithm applied for feature extractor across validation sets for predicting cardiac cause mortality. Table S11. Comprehensive metadata for CXR imaging parameters Figure S1. Flow chart of patient inclusion and exclusion in (A) TLCID, (B) ChestDR and (C) PLCO. CXR: chest X‐ray; TMUCIH, Tianjin Medical University Cancer Institute and Hospital; PLCO: The Prostate, Lung, Colorectal and Ovarian Cancer Screening Trial; TLCID: Tianjin Lung Cancer Imaging Dataset. Figure S2. Representative examples of chest X‐rays with pulmonary nodules of different diameters. (A)Pulmonary nodule with a diameter of less than 5 mm.(B) Pulmonary nodule with a diameter between [file MCO2-7-e70730-s001.docx]

**Supplementary Material**

**Predicting Pulmonary Nodules and All-Cause and Cause-Specific Mortality in High-Risk Adults Using Chest Radiography and Epidemiologic Characteristics: A Multicenter Cohort Study**

**Running title: Nodules and Mortality Prognosis from CXR**

**Title page**

Junxian Li^1^, Yuchen Xing^2^, Ximin Gao^2^, Ya Liu^3^, Liwen Zhang^4^, Yubei Huang^2^, Pengyu Zhang^1^, Zhaoxiang Ye^5^, Meng Wang^6^, Fengju Song^2^**^*^**

^1^Department of Blood Transfusion, Key Laboratory of Cancer Prevention and Therapy in Tianjin, National Clinical Research Center for Cancer, Tianjin's Clinical Research Center for Cancer, Tianjin Medical University Cancer Institute and Hospital, Tianjin Medical University, Tianjin 300060, China.

^2^Department of Cancer Epidemiology and Biostatistics, Tianjin Key Laboratory of Molecular Cancer Epidemiology, Clinical Research Center for Cancer, Key Laboratory of Cancer Prevention and Therapy, Tianjin's Clinical Research Center for Cancer, Tianjin Medical University Cancer Institute and Hospital, Tianjin Medical University, Tianjin 300060, China.

^3^Public Health Science and Engineering College, Tianjin University of Traditional Chinese Medicine, Tianjin 300060, China.

^4^Department of Epidemiology and Statistics, School of Public Health, Hebei Medical University, Hebei Key Laboratory of Environment and Human Health, Shijiazhuang 050017, China.

^5^Department of Radiology, National Clinical Research Centre for Cancer, Key Laboratory of Cancer Prevention and Therapy, Tianjin’s Clinical Research Center for Cancer, Tianjin Medical University Cancer Institute and Hospital, Tianjin Medical University, Tianjin 300060, China.

^6^Department of Lung Cancer, Key Laboratory of Cancer Prevention and Therapy, National Clinical Research Center for Cancer, Tianjin’s Clinical Research Center for Cancer, Tianjin Medical University Cancer Institute and Hospital, Tianjin Medical University, Tianjin 300060, China.

**^*^ Corresponding should be given to:** Fengju Song

Fengju Song, PhD, Professor, Department of Epidemiology and Biostatistics, Key Laboratory of Molecular Cancer Epidemiology, Tianjin, Key Laboratory of Breast Cancer Prevention and Therapy, Tianjin Medical University, Ministry of Education, National Clinical Research Center for Cancer, Tianjin’s Clinical Research Center for Cancer, Tianjin Medical University Cancer Institute and Hospital, Tianjin, 300060, China.

Tel.:++86 (0)2223372231; Fax:++86 (0)2223372231; E-mail address: [songfengju@163.com](mailto:songfengju@163.com)

**Supplementary Material I：** **Deep-Learning Backbone Structures Used as Feature Extractors**

1. **AlexNet**

AlexNet is a pioneering deep convolutional network that won the 2012 ImageNet Large-Scale Visual Recognition Challenge. It consists of five convolutional layers followed by three fully connected layers. ReLU activations replace the traditional tanh / sigmoid functions, markedly accelerating convergence. Local Response Normalization (LRN) and Dropout are applied to curb over-fitting, while random cropping and mirroring provide data augmentation that further enhances generalization.

1. **DenseNet-121**

DenseNet-121 is composed of multiple dense blocks in which every layer is connected to all preceding layers, enabling features to be fully integrated and propagated; these skip-connections are implemented by concatenation (rather than summation), so earlier feature maps are preserved intact. In addition, DenseNet-121 employs transition layers that perform dimensionality reduction and down-sampling of feature maps, linking one dense block to the next. The architecture begins with an input layer, followed by a 7×7 convolution and a 3×3 max-pooling stem; next come four iterations of “dense block then transition layer”; a global average-pooling layer follows; and the network terminates with a fully connected soft-max classifier. Its advantages stem from the full feature reuse governed by the growth-rate hyper-parameter 𝑘, which keeps the model compact; connecting each layer to all previous layers not only lowers the parameter count compared with equally deep plain convolutional neural networks (CNNs) but also mitigates gradient vanishing/exploding, allowing gradients to flow directly through the network. A bottleneck pattern (1×1 convolution followed by a 3×3 convolution) inside each composite layer additionally reduces memory footprint and FLOPs, making DenseNet-121 efficient during both training and inference.

1. **GoogLeNet**

GoogLeNet is built around the Inception module. Each module contains several parallel branches: convolution kernels of multiple receptive-field sizes (e.g., 1×1, 3×3, 5×5) and a max-pooling branch, whose outputs are concatenated along the channel dimension. By stacking nine such modules, GoogLeNet reaches a depth of 22 learnable layers. To mitigate vanishing gradients, two auxiliary soft-max classifiers are attached after specific Inception modules, injecting additional gradient signals during back-propagation. Thanks to its multi-scale feature extraction, dimensionality-reduction 1×1 convolutions, and global average pooling in place of fully connected layers, GoogLeNet achieves high recognition accuracy while keeping parameters and FLOPs low. Consequently, it offers a favorable trade-off between performance, efficiency, and generalization, and has become a strong baseline for large-scale image classification tasks.

1. **MnasNet-0.5**

MnasNet-0.5 is a lightweight convolutional neural network tailored for mobile and embedded platforms. Platform-aware neural architecture search (NAS)—an AutoML approach that explicitly optimizes the accuracy–latency trade-off on target hardware—yields its architecture. The model employs depthwise-separable convolutions and squeeze-and-excitation units to minimize FLOPs and parameter count while preserving competitive ImageNet classification accuracy. The network comprises a series of mobile inverted bottleneck (MBConv) blocks, each containing an expansion layer, a depthwise convolution, and a 1×1 point-wise projection. Exposed hyper-parameters—namely the width multiplier (α), depth multiplier (ρ), and dropout rate (p)—enable practitioners to fine-tune the network’s width, depth, and regularization strength to meet diverse resource budgets.

1. **MobileNet-V2**

MobileNet-V2 is a lightweight CNN tailored for mobile and embedded platforms. It replaces standard convolutions with depthwise-separable convolutions, thereby lowering parameter count and FLOPs. To preserve representational capacity, the network introduces inverted residual blocks with a linear bottleneck, which funnel information through narrow, linear projections before expansion. MobileNet-V2 contains ≈53 learnable layers and employs ReLU6 activation together with batch-normalization layers, which stabilize and accelerate training. Exposed hyper-parameters—namely the width multiplier (α), the input-resolution multiplier (ρ), and the dropout rate (p)—allow practitioners to tune the model’s width, spatial resolution, and regularization strength, achieving an optimal balance between accuracy and on-device latency.

1. **SqueezeNet-1.1**

SqueezeNet-1.1 is a compact convolutional neural network that substantially reduces parameter count while sustaining competitive ImageNet accuracy. It applies three design strategies—(i) replacing many 3×3 kernels with 1×1 kernels, (ii) decreasing the number of input channels to 3×3 filters via squeeze layers, and (iii) postponing down-sampling to later layers to enlarge activation maps. The core Fire module first squeezes feature maps with a 1×1 convolution and then expands them with a mixture of 1×1 and 3×3 convolutions, thereby lowering both parameters and FLOPs. SqueezeNet-1.1 fits ≈50 × fewer parameters than AlexNet and replaces fully connected layers with global-average pooling to further shrink model size. Compared with the original SqueezeNet-1.0, version 1.1 widens early layers and removes some pooling operations, delivering up to 2.4× higher throughput on mobile GPUs. Exposed hyper-parameters—the squeeze ratio, expand ratio, and the number of Fire modules—allow practitioners to adjust channel widths, receptive-field diversity, and depth to satisfy diverse accuracy-latency constraints.

1. **VGG-16**

VGG-16 is a seminal CNN introduced in the 2014 ILSVRC. It stacks 13 convolutional layers—all employing 3×3 kernels with stride 1 and same padding—and interleaves 2×2 max-pooling layers with stride 2 to down-sample feature maps. Three fully connected layers follow the convolutional backbone, bringing the total to 16 learnable layers. Each convolution is immediately followed by ReLU activation and batch normalization in modern re-implementations, which stabilises gradients and speeds up convergence.

The network hosts≈138 million parameters and incurs substantial FLOPs, yet its straightforward “deep-and-narrow” design delivers strong top-1 accuracy on ImageNet. Consequently, VGG-16 remains a widely used baseline and a reliable feature extractor for transfer learning, object detection, and style-transfer tasks. Practitioners typically tune the per-stage channel widths, the learning-rate schedule, the weight-decay coefficient (L2 regularization), and the dropout rate applied to the fully connected layers to balance generalization against overfitting and to adapt the model to specific compute budgets.

1. **ViT**

Vision Transformer (ViT) is a pure-Transformer architecture for image classification that first appears in 2020. It partitions each input image into non-overlapping patches (e.g., 16×16), flattens them, projects them through a shared linear layer, adds learned positional embeddings, and prepends a learnable classification token. The resulting token sequence passes through a standard Transformer encoder that applies multi-head self-attention and position-wise feed-forward networks to model long-range dependencies across the entire image.

By replacing locality-biased convolutions with global attention, ViT captures holistic context and shows strong scalability: when pre-trained on sufficiently large datasets and fine-tuned on downstream tasks, it matches or surpasses state-of-the-art convolutional networks while offering a simpler, uniform architecture. Key hyper-parameters—the patch size, hidden size (d_model), Transformer depth (L), number of attention heads (H), MLP hidden dimension (d_ff), and the dropout rate—jointly determine the accuracy–efficiency trade-off and are routinely tuned to accommodate diverse compute budgets and resolution requirements.

**Supplementary Material II：** **ML network structures applied for classifiers**

1. **Logistic Regression**

Logistic Regression is an extensively utilized statistical method for tackling binary classification problems. It models the probability of a given input belonging to a specific class by establishing a linear relationship between the input features and the log-odds of the target variable. Specifically, logistic regression utilizes the logistic function, also known as the sigmoid function, to transform the linear combination of input features into a probability value between 0 and 1. The function is expressed as:

$$P\left( y = 1 | X \right)= \frac{1}{\left( 1 + e^{-\left( \beta^{0}+ \beta^{1}x^{1}+ \beta^{2}x^{2}+ \ldots+ \betaₙxₙ \right)} \right)}$$

where $\left( y = 1 | X \right)$ is the probability that the instance belongs to class 1, X represents the input features, and β₀, β₁, ..., βₙ are the parameters to be estimated.

During the training phase, logistic regression employs maximum likelihood estimation (MLE) to identify the optimal parameters that maximize the likelihood of the observed data under the model. This process relies on an iterative optimization algorithm, such as gradient descent, which aims to minimize the cost function. In this context, the cost function is defined as the negative log-likelihood.

For this study, the logistic regression model is implemented with the following hyperparameters: the regularization method was set to L2 (ridge) to prevent overfitting; the regularization strength (C) was set to 1.0, balancing bias and variance; the solver used for optimization was “liblinear”, which is well suited to smaller datasets; and the maximum number of iterations for the solver was set to 100 to ensure convergence. These settings aim to achieve efficient training while mitigating overfitting.

1. **NaiveBayes**

Naive Bayes is a probabilistic classifier based on Bayes' theorem that assumes conditional independence between features given the class label. It is widely used in text classification and other applications where this assumption is reasonably satisfied. Naive Bayes is highly scalable, requiring a number of parameters linear to the number of features in the learning problem. In the model development and tuning phase, parameters such as “alpha” (the Laplace-smoothing parameter) and “priors” (class prior probabilities) can be adjusted to enhance performance.

The training process of Naive Bayes first estimates the prior probability $P\left( y \right)$, which represents the proportion of samples belonging to each class in the training set. It then computes the conditional probability$P\left( x_{i} | y \right)$ of each feature value given the class. For discrete features, these conditional probabilities derive from frequency statistics; for continuous features, the model assumes a Gaussian distribution and calculates the probability density using the mean and standard deviation of that feature within each class. To avoid zero-probability issues caused by unseen feature values, it applies Laplace smoothing with the parameter 𝛼 ensuring robustness even when rare feature values are present.

During the prediction phase, Naive Bayes combines the prior and conditional probabilities to calculate the posterior probability for each class:

$$P\left( y | x \right)\propto P\left( y \right)\prod P\left( x_{i} | y \right)$$

Finally, the class with the highest posterior probability is selected as the prediction result. Implementations such as GaussianNB, MultinomialNB, or BernoulliNB are tailored for continuous, discrete, and binary features respectively.

1. **Support Vector Machine**

Support Vector Machine (SVM) is a supervised learning model that tackles classification and regression tasks. SVM constructs one or more hyperplanes in a high-dimensional space to separate different classes, and seeks the hyperplane that maximizes the margin between them. SVM performs well in high-dimensional spaces and supports various kernel functions—linear, polynomial, and radial basis function (RBF). Key hyperparameters include “C” (regularization parameter), “kernel” (type of kernel), and “gamma” (the bandwidth parameter for RBF kernel).

The goal of training an SVM is to maximize the margin between classes while minimizing classification error. The algorithm first scales the input features to ensure numerical stability. For linearly separable data, SVM solves the following optimization problem:

$$\begin{aligned} min\frac{1}{2}\parallel w\parallel^{2} \\ \text{ } \end{aligned}$$

$$subject to y_{i}\left( w^{T}x_{i}+b \right)\geq1$$

Here, $\parallel w\parallel$ represents the norm of the hyperplane's normal vector, and $y_{i}$is the class label of the sample. The goal is to maximize the margin while ensuring all samples are correctly classified.

For non-linearly separable data, SVM uses kernel functions (such as the RBF kernel, linear kernel, or polynomial kernel) to map the original data into a higher-dimensional feature space, where a linear hyperplane can be found. In this case, slack variables$\xi_{i}$ and the regularization parameter $C$ handle misclassified samples, yielding

$$min\frac{1}{2}\parallel w\parallel^{2}+C\sum_{i=1}^{n} \xi_{i}$$

$$\text{subject to }y_{i}\left( w^{T}\phi\left( x_{i} \right)+b \right)\geq1-\xi_{i},\xi_{i}\geq0$$

Here, $\phi\left( x_{i} \right)$represents the mapping function that projects the original data into a higher-dimensional space. The parameter $C$ controls the trade-off between maximizing the margin and minimizing the classification error.

During training, support vectors—samples that lie on or inside the margin—determine the final hyperplane. Solving the quadratic programming problem yields the parameters $w$ and $b$. After training, the model predicts the class of a new sample $x$ using

$$f(x)=w^{T}\phi(x)+b$$

If $f\left( x \right)>0$, the sample is assigned to the positive class; otherwise it is assigned to the negative class.

1. **Random Forest**

Random Forest is an ensemble learning method that constructs a multitude of decision trees during training and outputs either the class corresponding to the mode of individual-treepredictions (classification) or the mean of these predictions (regression). lt successfully handlesboth classification and regression tasks. Random Forest reduces overfitting by averaging thepredictions of multiple trees, each trained on bootstrap samples of the data and evaluatedwith randomly selected feature subsets.

The training process in Random Forest model primarily relies on two key techniques: bootstrap aggregation (commonly referred to as bagging) and random feature selection. During training, the algorithm randomly extracts subsets of samples with replacement from the original dataset (referred to as “bootstrap samples”) to generate independent training datasets for each decision tree, thereby introducing diversity among the trees. For each tree, a subset of features is randomly selected at each split to identify the best split point. This approach effectively reduces the correlation between decision trees and enhances the model’s stability and generalization ability.

In this study, the hyperparameters for the Random Forest model are configured as follows: the number of decision trees in the forest (n_estimators) is set to 100; the number of features considered for splitting at each node (max_features) is set to “sqrt” which selects the square root of the total number of features; the maximum depth of each tree (max_depth) is limited to 10 to prevent overfitting; the minimum number of samples required to split a node (min_samples_split) is set to 2; and the minimum number of samples required to form a leaf node (min_samples_leaf) is set to 1.

1. **XGBoost**

XGBoost (eXtreme Gradient Boosting) is an optimized, distributed gradient-boosting library that remains highly efficient, flexible, and portable. It implements parallel tree boosting, which solves many data-science tasks quickly and accurately. Model training progressively optimizes an objective function to minimize prediction error. This objective combines (1) a loss term—typically the logarithmic loss for classification or the squared error for regression—that quantifies the discrepancy between predicted and observed values, and (2) a regularization term that constrains tree depth and leaf weights to suppress overfitting. At each iteration, the algorithm grows a new regression tree that fits a share of the residuals left by the ensemble. Unlike classical gradient boosting, XGBoost integrates both first- and second-order gradient information, enabling more precise split finding and faster convergence. To enhance generalization, hyper-parameters such as max_depth (here set to 8) limit tree complexity, subsample controls the row-sampling ratio, and colsample_bytree governs column sub-sampling. Additional settings—including the learning-rate parameter η, the minimum loss-reduction threshold γ required for a split, and the L1/L2 regularization weights α and λ—provide fine-grained control over the bias–variance trade-off.

1. **LightGBM**

LightGBM (Light Gradient Boosting Machine) is a distributed, tree-based gradient-boosting framework that handles large-scale data with high training speed and efficiency. Training starts by initializing a simple prediction—typically the mean of the target variable—and defining an objective function that combines (1) a loss term measuring prediction error and (2) a regularization term that constrains model complexity to prevent overfitting. The algorithm iteratively grows an ensemble of decision trees; each newly added tree fits the residuals left by the previous iterations, thereby reducing overall error.

During training, LightGBM employs a histogram-based splitting strategy that discretizes continuous features into fixed bins. At every split, the algorithm builds histograms and rapidly identifies the optimal split point by maximizing information gain. It follows a leaf-wise growth policy, always expanding the leaf with the highest gain to improve accuracy.

Hyper-parameters are carefully tuned to balance bias and variance: num_leaves controls model complexity, max_depth = 8 constrains tree depth, and learning_rate = 0.1 moderates the contribution of each boosting round. In addition, feature_fraction = 0.8 and bagging_fraction = 0.8 randomize feature and sample subsets, respectively, which introduces stochasticity and reduces overfitting risk.

1. **GradientBoosting**

Gradient Boosting is an ensemble learning technique that builds models in a stage-wise manner and optimizes an arbitrary differentiable loss function, making it applicable to both classification and regression. Training starts by initializing a baseline predictor—typically the logarithmic odds for binary classification or the mean target value for regression. At every iteration, the algorithm computes the residuals, which correspond to the negative gradient of the loss, and fits a shallow decision tree to these residuals. The tree depth is limited to max_depth = 4, balancing model expressiveness and overfitting risk. The tree’s predictions are then scaled by a learning rate (learning_rate = 0.1) before being added to the ensemble. This process repeats until n_estimators = 200 trees are built, enabling the model to capture complex, nonlinear patterns. To enhance generalization, the algorithm samples subsample = 0.8 of the training data at each boosting step, injecting randomness that further curbs overfitting.

1. **AdaBoost**

AdaBoost (Adaptive Boosting) is an ensemble algorithm that sequentially combines many weak learners—typically decision stumps—into a strong classifier. Training starts by assigning equal weights to all samples and fitting the first weak learner to minimize the weighted classification error. After each iteration, the algorithm increases the weights of misclassified samples and decreases those of correctly classified ones, forcing the next learner to focus on difficult cases. The weight of the t-th learner is

$$\alpha_{t}= \backslash frac12 \backslash ln!\left. \left( \backslash frac\left\{ 1-\varepsilon_{t} \right\}\left\{ \varepsilon_{t} \right\} \right. \right)$$

where $\varepsilon_{t}$ denotes its weighted error rate. Final predictions are obtained by a weighted majority vote (or weighted sum for regression) of all learners, which effectively reduces the exponential loss and improves generalization.

1. **Multilayer Perceptron**

A Multilayer Perceptron (MLP) is a supervised-learning model that approximates nonlinear functions for classification or regression. In contrast with logistic regression, an MLP inserts one or more nonlinear hidden layers between the input and output layers, enabling it to capture complex feature interactions. Training iteratively adjusts the network weights with back-propagation to minimize a chosen loss. During the forward pass, each layer applies an affine transformation followed by a nonlinear activation, producing high-dimensional representations that culminate in the output layer’s prediction. The model then evaluates the prediction error with a cross-entropy loss for classification (or a mean-squared error for regression). In the backward pass, the chain rule computes gradients of the loss with respect to all parameters, and the Adam optimizer updates the weights, progressively reducing the loss. Because the loss surface is non-convex, different random initializations can lead to distinct local minima and hence variable validation accuracy. Training proceeds for a fixed number of epochs or until a convergence criterion is met, yielding a network capable of accurate predictions.

**Table Legend of** **the Supplementary file**

**Table S1.** Performance comparison of mainstream DL algorithm applied for feature extractor across validation sets.

**Table S2.** Performance comparison of mainstream ML algorithm applied for classifiers across validation sets.

**Table S3.** Performance comparison of mainstream DL algorithm applied for feature extractor across validation sets for predicting all-cause mortality.

**Table S4.** Performance comparison of mainstream DL algorithm applied for feature extractor across validation sets for predicting COPD -cause mortality.

**Table S5.** Performance comparison of mainstream DL algorithm applied for feature extractor across validation sets for predicting lung cancer -cause mortality.

**Table S6.** Performance comparison of mainstream DL algorithm applied for feature extractor across validation sets for predicting cardiac-cause mortality.

**Table S7.** Performance comparison of mainstream ML algorithm applied for feature extractor across validation sets for predicting all-cause mortality.

**Table S8.** Performance comparison of mainstream ML algorithm applied for feature extractor across validation sets for predicting COPD-cause mortality.

**Table S9.** Performance comparison of mainstream ML algorithm applied for feature extractor across validation sets for predicting lung cancer -cause mortality.

**Table S10.** Performance comparison of mainstream ML algorithm applied for feature extractor across validation sets for predicting cardiac cause mortality.

**Table S11.** Comprehensive metadata for CXR imaging parameters

**Figure Legend of** **the Supplementary file**

**Figure S1.** Flow chart of patient inclusion and exclusion in (A) TLCID, (B) ChestDR and (C) PLCO. CXR: chest X-ray; TMUCIH, Tianjin Medical University Cancer Institute and Hospital; PLCO: The Prostate, Lung, Colorectal and Ovarian Cancer Screening Trial; TLCID: Tianjin Lung Cancer Imaging Dataset.

**Figure S2.** Representative examples of chest X-rays with pulmonary nodules of different diameters. **(A)** Pulmonary nodule with a diameter of less than 5 mm.**(B)** Pulmonary nodule with a diameter between 5 mm and 10 mm.**(C)** Pulmonary nodule with a diameter greater than 10 mm.

**Table S1.** Performance comparison of mainstream DL algorithm applied for feature extractor across validation sets.

|  | TLCID | | | |  | ChestDR | | | |
| --- | --- | --- | --- | --- | --- | --- | --- | --- | --- |
| Model | AUC (95% CI) | Accuracy | Sensitivity | Specificity |  | AUC (95% CI) | Accuracy | Sensitivity | Specificity |
| AlexNet | 0.894 (0.883-0.905) | 0.898 | 0.761 | 0.903 |  | 0.718 (0.698-0.739) | 0.142 | 0.998 | 0.005 |
| DenseNet-121 | 0.903 (0.856-0.946) | 0.999 | 0.469 | 1.000 |  | 0.673 (0.550-0.778) | 0.999 | 0.990 | 0.010 |
| GoogLeNet | 0.881 (0.868-0.892) | 0.913 | 0.733 | 0.920 |  | 0.709 (0.688-0.729) | 0.147 | 0.998 | 0.011 |
| MnasNet-0.5 | 0.882 (0.869-0.893) | 0.919 | 0.723 | 0.927 |  | 0.729 (0.708-0.750) | 0.153 | 0.997 | 0.019 |
| MobileNet-V2 | 0.891 (0.879-0.902) | 0.898 | 0.756 | 0.904 |  | 0.708 (0.689-0.728) | 0.144 | 0.998 | 0.008 |
| SqueezeNet-1.1 | 0.881 (0.869-0.892) | 0.911 | 0.732 | 0.918 |  | 0.726 (0.708-0.744) | 0.162 | 0.998 | 0.029 |
| VGG-16 | 0.890 (0.877-0.901) | 0.908 | 0.743 | 0.914 |  | 0.748 (0.728-0.767) | 0.150 | 1.000 | 0.015 |
| ViT | 0.866 (0.854-0.879) | 0.912 | 0.711 | 0.920 |  | 0.643 (0.620-0.667) | 0.215 | 0.953 | 0.097 |

**Table S2.** Performance comparison of mainstream ML algorithm applied for classifiers across validation sets.

|  | TLCID | | | |  | ChestDR | | | |
| --- | --- | --- | --- | --- | --- | --- | --- | --- | --- |
| Model | AUC (95% CI) | Accuracy | Sensitivity | Specificity |  | AUC (95% CI) | Accuracy | Sensitivity | Specificity |
| LR | 0.933(0.919 - 0.946) | 0.857 | 0.837 | 0.876 |  | 0.805(0.787 - 0.821) | 0.712 | 0.756 | 0.705 |
| NaiveBayes | 0.805(0.780 - 0.829) | 0.745 | 0.671 | 0.813 |  | 0.760(0.740 - 0.778) | 0.659 | 0.77 | 0.641 |
| SVM | 0.717(0.688 - 0.746) | 0.713 | 0.483 | 0.926 |  | 0.596(0.568 - 0.622) | 0.823 | 0.364 | 0.896 |
| RandomForest | 0.705(0.681 - 0.729) | 0.699 | 0.524 | 0.862 |  | 0.660(0.640 - 0.680) | 0.849 | 0.281 | 0.939 |
| XGBoost | 0.857(0.835 - 0.877) | 0.794 | 0.703 | 0.878 |  | 0.765(0.745 - 0.784) | 0.705 | 0.704 | 0.705 |
| LightGBM | 0.828(0.806 - 0.850) | 0.747 | 0.714 | 0.779 |  | 0.746(0.725 - 0.767) | 0.678 | 0.722 | 0.671 |
| GradientBoosting | 0.809(0.785 - 0.833) | 0.777 | 0.849 | 0.71 |  | 0.719(0.699 - 0.739) | 0.608 | 0.749 | 0.585 |
| AdaBoost | 0.923(0.908 - 0.937) | 0.855 | 0.839 | 0.87 |  | 0.767(0.748 - 0.785) | 0.755 | 0.583 | 0.782 |
| MLP | 0.922(0.906 - 0.936) | 0.844 | 0.854 | 0.835 |  | 0.789(0.771 - 0.806) | 0.696 | 0.758 | 0.686 |

**Table S3.** Performance comparison of mainstream DL algorithm applied for feature extractor across validation sets for predicting all-cause mortality.

| Model | AUC (95% CI) | Accuracy | Sensitivity | Specificity |
| --- | --- | --- | --- | --- |
| 1 year |  |  |  |  |
| AlexNet | 0.794 (0.782 - 0.806) | 0.782 | 0.722 | 0.792 |
| DenseNet-121 | 0.798 (0.786 - 0.810) | 0.788 | 0.753 | 0.796 |
| GoogLeNet | 0.788 (0.776 - 0.800) | 0.799 | 0.718 | 0.787 |
| MnasNet-0.5 | 0.795 (0.783 - 0.807) | 0.784 | 0.728 | 0.793 |
| MobileNet-V2 | 0.791 (0.780 - 0.803) | 0.781 | 0.721 | 0.789 |
| SqueezeNet-1.1 | 0.787 (0.775 - 0.799) | 0.778 | 0.712 | 0.786 |
| VGG-16 | 0.799 (0.787 - 0.811) | 0.792 | 0.775 | 0.798 |
| ViT | 0.793 (0.781 - 0.805) | 0.783 | 0.731 | 0.791 |
| 2 years |  |  |  |  |
| AlexNet | 0.782 (0.771-0.792) | 0.771 | 0.711 | 0.791 |
| DenseNet-121 | 0.786 (0.775-0.796) | 0.782 | 0.735 | 0.789 |
| GoogLeNet | 0.788 (0.778-0.798) | 0.784 | 0.745 | 0.787 |
| MnasNet-0.5 | 0.781 (0.770-0.791) | 0.772 | 0.715 | 0.789 |
| MobileNet-V2 | 0.784 (0.773-0.794) | 0.779 | 0.721 | 0.790 |
| SqueezeNet-1.1 | 0.785 (0.775-0.795 | 0.780 | 0.729 | 0.788 |
| VGG-16 | 0.789 (0.778-0.799) | 0.786 | 0.749 | 0.792 |
| ViT | 0.780 (0.769-0.790) | 0.770 | 0.739 | 0.0785 |
| 3 years |  |  |  |  |
| AlexNet | 0.772 (0.761-0.782) | 0.769 | 0.732 | 0.782 |
| DenseNet-121 | 0.773 (0.762-0.783) | 0.770 | 0.725 | 0.784 |
| GoogLeNet | 0.761 (0.750-0.771) | 0.762 | 0.712 | 0.775 |
| MnasNet-0.5 | 0.769 (0.758-0.779) | 0.765 | 0.728 | 0.781 |
| MobileNet-V2 | 0.774 (0.763-0.784) | 0.771 | 0.719 | 0.785 |
| SqueezeNet-1.1 | 0.758 (0.747-0.768) | 0.760 | 0.702 | 0.778 |
| VGG-16 | 0.771 (0.760-0.781) | 0.768 | 0.701 | 0.783 |
| ViT | 0.768 (0.757-0.778) | 0.763 | 0.722 | 0.780 |
| 5 years |  |  |  |  |
| AlexNet | 0.722 (0.712-0.732) | 0.732 | 0.682 | 0.762 |
| DenseNet-121 | 0.724 (0.714-0.734) | 0.734 | 0.685 | 0.764 |
| GoogLeNet | 0.754 (0.744-0.764) | 0.748 | 0.712 | 0.755 |
| MnasNet-0.5 | 0.723 (0.713-0.733) | 0.733 | 0.681 | 0.763 |
| MobileNet-V2 | 0.720 (0.710-0.730) | 0.729 | 0.679 | 0.759 |
| SqueezeNet-1.1 | 0.757 (0.747-0.767) | 0.751 | 0.715 | 0.757 |
| VGG-16 | 0.725 (0.715-0.735) | 0.736 | 0.692 | 0.765 |
| ViT | 0.756 (0.746-0.766) | 0.749 | 0.713 | 0.758 |
| 12 years |  |  |  |  |
| AlexNet | 0.692 (0.682-0.702) | 0.702 | 0.632 | 0.702 |
| DenseNet-121 | 0.694 (0.684-0.704) | 0.701 | 0.638 | 0.704 |
| GoogLeNet | 0.689 (0.679-0.699) | 0.699 | 0.628 | 0.701 |
| MnasNet-0.5 | 0.693 (0.683-0.703) | 0.700 | 0.635 | 0.703 |
| MobileNet-V2 | 0.692 (0.682-0.702) | 0.701 | 0.634 | 0.702 |
| SqueezeNet-1.1 | 0.691 (0.681-0.701) | 0.699 | 0.629 | 0.701 |
| VGG-16 | 0.695 (0.685-0.705) | 0.703 | 0.641 | 0.705 |
| ViT | 0.689 (0.679-0.699) | 0.699 | 0.625 | 0.700 |

**Table S4.** Performance comparison of mainstream DL algorithm applied for feature extractor across validation sets for predicting COPD -cause mortality.

| Model | AUC (95% CI) | Accuracy | Sensitivity | Specificity |
| --- | --- | --- | --- | --- |
| 1 year |  |  |  |  |
| AlexNet | 0.782 (0.715 - 0.792) | 0.723 | 0.681 | 0.742 |
| DenseNet-121 | 0.642 (0.612 - 0.678) | 0.712 | 0.695 | 0.738 |
| GoogLeNet | 0.764 (0.721 - 0.787) | 0.715 | 0.668 | 0.738 |
| MnasNet-0.5 | 0.732 (0.698 - 0.778) | 0.719 | 0.674 | 0.736 |
| MobileNet-V2 | 0.682 (0.632 - 0.725) | 0.706 | 0.635 | 0.729 |
| SqueezeNet-1.1 | 0.754 (0.715 - 0.776) | 0.718 | 0.682 | 0.732 |
| VGG-16 | 0.723 (0.692 - 0.749) | 0.727 | 0.672 | 0.745 |
| ViT | 0.734 (0.704 - 0.767) | 0.721 | 0.679 | 0.739 |
| 2 years |  |  |  |  |
| AlexNet | 0.752 (0.712 - 0.788) | 0.728 | 0.732 | 0.749 |
| DenseNet-121 | 0.782 (0.741 - 0.792) | 0.745 | 0.756 | 0.752 |
| GoogLeNet | 0.723 (0.682 - 0.767) | 0.715 | 0.712 | 0.734 |
| MnasNet-0.5 | 0.728 (0.698 - 0.769) | 0.719 | 0.724 | 0.738 |
| MobileNet-V2 | 0.718 (0.693 - 0.749 | 0.711 | 0.728 | 0.732 |
| SqueezeNet-1.1 | 0.727 (0.714 - 0.754) | 0.718 | 0.735 | 0.729 |
| VGG-16 | 0.774 (0.745 - 0.789) | 0.752 | 0.742 | 0.758 |
| ViT | 0.762 (0.742 - 0.783) | 0.739 | 0.721 | 0.749 |
| 3 years |  |  |  |  |
| AlexNet | 0.692 (0.672 - 0.732) | 0.715 | 0.704 | 0.729 |
| DenseNet-121 | 0.698 (0.682 - 0.738) | 0.722 | 0.729 | 0.736 |
| GoogLeNet | 0.704 (0.682 - 0.745) | 0.712 | 0.698 | 0.728 |
| MnasNet-0.5 | 0.689 (0.675 - 0.726) | 0.708 | 0.707 | 0.724 |
| MobileNet-V2 | 0.684 (0.672 - 0.736) | 0.718 | 0.719 | 0.734 |
| SqueezeNet-1.1 | 0.691 (0.674 - 0.728) | 0.706 | 0.713 | 0.721 |
| VGG-16 | 0.702 (0.692 - 0.739) | 0.724 | 0.721 | 0.742 |
| ViT | 0.698 (0.685 - 0.734) | 0.716 | 0.716 | 0.732 |
| 5 years |  |  |  |  |
| AlexNet | 0.689 (0.672 - 0.712) | 0.712 | 0.682 | 0.724 |
| DenseNet-121 | 0.732 (0.712 - 0.759) | 0.737 | 0.732 | 0.749 |
| GoogLeNet | 0.701 (0.678 - 0.729) | 0.718 | 0.698 | 0.734 |
| MnasNet-0.5 | 0.706 (0.685 - 0.734) | 0.722 | 0.716 | 0.739 |
| MobileNet-V2 | 0.695 (0.682 - 0.718) | 0.715 | 0.708 | 0.729 |
| SqueezeNet-1.1 | 0.698 (0.684 - 0.724) | 0.726 | 0.722 | 0.742 |
| VGG-16 | 0.709 (0.692 - 0.738) | 0.732 | 0.727 | 0.752 |
| ViT | 0.704 (0.687 - 0.728) | 0.725 | 0.712 | 0.738 |
| 12 years |  |  |  |  |
| AlexNet | 0.652 (0.642 - 0.687) | 0.698 | 0.623 | 0.702 |
| DenseNet-121 | 0.643 (0.632 - 0.664) | 0.695 | 0.638 | 0.704 |
| GoogLeNet | 0.634 (0.626 - 0.658) | 0.687 | 0.615 | 0.692 |
| MnasNet-0.5 | 0.648 (0.635 - 0.674) | 0.694 | 0.628 | 0.698 |
| MobileNet-V2 | 0.655 (0.642 - 0.682) | 0.701 | 0.632 | 0.705 |
| SqueezeNet-1.1 | 0.626 (0.618 - 0.652) | 0.684 | 0.621 | 0.691 |
| VGG-16 | 0.647 (0.638 - 0.672) | 0.699 | 0.631 | 0.701 |
| ViT | 0.632 (0.625 - 0.657) | 0.689 | 0.617 | 0.693 |

**Table S5.** Performance comparison of mainstream DL algorithm applied for feature extractor across validation sets for predicting lung cancer -cause mortality.

| Model | AUC (95% CI) | Accuracy | Sensitivity | Specificity |
| --- | --- | --- | --- | --- |
| 1 year |  |  |  |  |
| AlexNet | 0.692 (0.642 - 0.742) | 0.783 | 0.559 | 0.785 |
| DenseNet-121 | 0.687 (0.638 - 0.736) | 0.781 | 0.685 | 0.782 |
| GoogLeNet | 0.689 (0.639 - 0.739) | 0.782 | 0.497 | 0.786 |
| MnasNet-0.5 | 0.742 (0.716 - 0.768) | 0.791 | 0.498 | 0.792 |
| MobileNet-V2 | 0.743 (0.718 - 0.768) | 0.792 | 0.685 | 0.794 |
| SqueezeNet-1.1 | 0.691 (0.641 - 0.741) | 0.784 | 0.499 | 0.788 |
| VGG-16 | 0.703 (0.659 - 0.747) | 0.788 | 0.622 | 0.790 |
| ViT | 0.748 (0.721 - 0.775) | 0.793 | 0.686 | 0.794 |
| 2 years |  |  |  |  |
| AlexNet | 0.708 (0.651 - 0.765) | 0.774 | 0.144 | 0.782 |
| DenseNet-121 | 0.704 (0.651 - 0.757) | 0.773 | 0.099 | 0.780 |
| GoogLeNet | 0.702 (0.654 - 0.750) | 0.772 | 0.078 | 0.779 |
| MnasNet-0.5 | 0.709 (0.652 - 0.766) | 0.775 | 0.143 | 0.783 |
| MobileNet-V2 | 0.707 (0.654 - 0.760) | 0.773 | 0.178 | 0.781 |
| SqueezeNet-1.1 | 0.697 (0.643 - 0.751) | 0.771 | 0.167 | 0.778 |
| VGG-16 | 0.712 (0.656 - 0.768) | 0.776 | 0.111 | 0.784 |
| ViT | 0.713 (0.667 - 0.759) | 0.777 | 0.168 | 0.785 |
| 3 years |  |  |  |  |
| AlexNet | 0.719 (0.689 - 0.749) | 0.762 | 0.013 | 0.771 |
| DenseNet-121 | 0.717 (0.687 - 0.747) | 0.761 | 0.013 | 0.771 |
| GoogLeNet | 0.716 (0.688 - 0.744) | 0.761 | 0.018 | 0.768 |
| MnasNet-0.5 | 0.718 (0.689 - 0.747) | 0.762 | 0.013 | 0.772 |
| MobileNet-V2 | 0.724 (0.693 - 0.755) | 0.765 | 0.009 | 0.773 |
| SqueezeNet-1.1 | 0.717 (0.684 - 0.750) | 0.762 | 0.009 | 0.770 |
| VGG-16 | 0.721 (0.692 - 0.750) | 0.764 | 0.013 | 0.774 |
| ViT | 0.719 (0.690 - 0.748) | 0.763 | 0.009 | 0.771 |
| 5 years |  |  |  |  |
| AlexNet | 0.675 (0.659 - 0.691) | 0.748 | 0.001 | 0.752 |
| DenseNet-121 | 0.672 (0.658 - 0.686) | 0.746 | 0.002 | 0.750 |
| GoogLeNet | 0.635 (0.619 - 0.651) | 0.734 | 0.004 | 0.740 |
| MnasNet-0.5 | 0.608 (0.594 - 0.622) | 0.726 | 0.004 | 0.732 |
| MobileNet-V2 | 0.675 (0.660 - 0.690) | 0.747 | 0.001 | 0.751 |
| SqueezeNet-1.1 | 0.624 (0.609 - 0.639) | 0.732 | 0.007 | 0.738 |
| VGG-16 | 0.644 (0.628 - 0.660) | 0.739 | 0.002 | 0.743 |
| ViT | 0.655 (0.640 - 0.670) | 0.742 | 0.004 | 0.746 |
| 12 years |  |  |  |  |
| AlexNet | 0.526 (0.512 - 0.540) | 0.847 | 0.108 | 0.862 |
| DenseNet-121 | 0.517 (0.504 - 0.530) | 0.843 | 0.104 | 0.859 |
| GoogLeNet | 0.483 (0.470 - 0.496) | 0.837 | 0.082 | 0.853 |
| MnasNet-0.5 | 0.495 (0.482 - 0.508) | 0.841 | 0.093 | 0.856 |
| MobileNet-V2 | 0.528 (0.514 - 0.542) | 0.849 | 0.113 | 0.863 |
| SqueezeNet-1.1 | 0.505 (0.493 - 0.517) | 0.839 | 0.099 | 0.857 |
| VGG16 | 0.534 (0.519 - 0.549) | 0.852 | 0.126 | 0.868 |
| ViT | 0.469 (0.456 - 0.482) | 0.834 | 0.079 | 0.851 |

**Table S6.** Performance comparison of mainstream DL algorithm applied for feature extractor across validation sets for predicting cardiac-cause mortality.

| Model | AUC (95% CI) | Accuracy | Sensitivity | Specificity |
| --- | --- | --- | --- | --- |
| 1 year |  |  |  |  |
| AlexNet | 0.685 (0.524 - 0.857) | 0.872 | 0.520 | 0.885 |
| DenseNet-121 | 0.681 (0.474 - 0.849) | 0.869 | 0.560 | 0.882 |
| GoogLeNet | 0.684 (0.490 - 0.857) | 0.868 | 0.440 | 0.880 |
| Mnasnet-0.5 | 0.840 (0.718 - 0.941) | 0.895 | 0.600 | 0.906 |
| MobileNet-V2 | 0.860 (0.749 - 0.948) | 0.902 | 0.520 | 0.912 |
| SqueezeNet-1.1 | 0.680 (0.500 - 0.848) | 0.867 | 0.480 | 0.878 |
| VGG-16 | 0.743 (0.356 - 1.000) | 0.881 | 0.480 | 0.893 |
| ViT | 0.883 (0.777 - 0.958) | 0.908 | 0.680 | 0.918 |
| 2 years |  |  |  |  |
| AlexNet | 0.721 (0.636 - 0.807) | 0.847 | 0.083 | 0.862 |
| DenseNet-121 | 0.715 (0.631 - 0.796) | 0.843 | 0.120 | 0.858 |
| GoogLeNet | 0.706 (0.619 - 0.789) | 0.839 | 0.037 | 0.853 |
| MnasNet-0.5 | 0.707 (0.626 - 0.793) | 0.841 | 0.083 | 0.856 |
| MobileNet-V2 | 0.711 (0.627 - 0.784) | 0.842 | 0.065 | 0.857 |
| SqueezeNet-1.1 | 0.707 (0.625 - 0.787) | 0.840 | 0.083 | 0.855 |
| VGG-16 | 0.706 (0.615 - 0.787) | 0.838 | 0.028 | 0.854 |
| ViT | 0.705 (0.615 - 0.791) | 0.844 | 0.093 | 0.860 |
| 3 years |  |  |  |  |
| AlexNet | 0.724 (0.676 - 0.774) | 0.831 | 0.034 | 0.843 |
| DenseNet-121 | 0.719 (0.668 - 0.773) | 0.828 | 0.019 | 0.840 |
| GoogLeNet | 0.714 (0.655 - 0.770) | 0.826 | 0.008 | 0.838 |
| MnasNet-0.5 | 0.725 (0.672 - 0.777) | 0.832 | 0.008 | 0.844 |
| MobileNet-V2 | 0.720 (0.669 - 0.771) | 0.829 | 0.015 | 0.841 |
| SqueezeNet-1.1 | 0.698 (0.645 - 0.751) | 0.824 | 0.023 | 0.836 |
| VGG-16 | 0.720 (0.668 - 0.770) | 0.830 | 0.023 | 0.836 |
| ViT | 0.706 (0.650 - 0.750) | 0.825 | 0.004 | 0.839 |
| 5 years |  |  |  |  |
| AlexNet | 0.662 (0.629 - 0.693) | 0.814 | 0.002 | 0.823 |
| DenseNet-121 | 0.676 (0.643 - 0.711) | 0.819 | 0.002 | 0.827 |
| GoogLeNet | 0.676 (0.639 - 0.705) | 0.818 | 0.005 | 0.826 |
| MnasNe-t0.5 | 0.662 (0.627 - 0.694) | 0.813 | 0.002 | 0.822 |
| MobileNet-V2 | 0.669 (0.634 - 0.706) | 0.816 | 0.003 | 0.824 |
| SqueezeNet-1.1 | 0.651 (0.615 - 0.683) | 0.809 | 0.005 | 0.818 |
| VGG-16 | 0.691 (0.656 - 0.723) | 0.823 | 0.003 | 0.832 |
| ViT | 0.664 (0.630 - 0.695) | 0.815 | 0.005 | 0.824 |
| 12 years |  |  |  |  |
| AlexNet | 0.417 (0.399 - 0.436) | 0.792 | 0.042 | 0.855 |
| DenseNet-121 | 0.445 (0.426 - 0.462) | 0.818 | 0.082 | 0.892 |
| GoogLeNet | 0.394 (0.377 - 0.411) | 0.786 | 0.035 | 0.852 |
| MnasNet-0.5 | 0.424 (0.405 - 0.441) | 0.802 | 0.068 | 0.866 |
| MobileNet-V2 | 0.718 (0.682 - 0.750) | 0.809 | 0.075 | 0.881 |
| SqueezeNet-1.1 | 0.403 (0.385 - 0.421) | 0.791 | 0.038 | 0.857 |
| VGG-16 | 0.417 (0.401 - 0.435) | 0.796 | 0.058 | 0.861 |
| ViT | 0.401 (0.383 - 0.418) | 0.788 | 0.036 | 0.854 |

**Table S7.** Performance comparison of mainstream ML algorithm applied for feature extractor across validation sets for predicting all-cause mortality.

| Model | AUC (95% CI) | Accuracy | Sensitivity | Specificity |
| --- | --- | --- | --- | --- |
| 1 year |  |  |  |  |
| LR | 0.655(0.596 - 0.713) | 0.652 | 0.583 | 0.652 |
| NaiveBayes | 0.639(0.580 - 0.698) | 0.784 | 0.458 | 0.784 |
| SVM | 0.928(0.889 - 0.966) | 0.936 | 0.823 | 0.936 |
| RandomForest | 0.920(0.884 - 0.956) | 0.996 | 0.542 | 0.997 |
| XGBoost | 0.639(0.586 - 0.692) | 0.959 | 0.167 | 0.960 |
| LightGBM | 0.546(0.509 - 0.582) | 0.963 | 0.115 | 0.964 |
| GradientBoosting | 0.550(0.515 - 0.584) | 0.965 | 0.115 | 0.966 |
| AdaBoost | 0.666(0.613 - 0.718) | 0.679 | 0.562 | 0.680 |
| MLP | 0.659(0.601 - 0.717) | 0.602 | 0.687 | 0.602 |
| 2 years |  |  |  |  |
| LR | 0.707(0.679 - 0.733) | 0.635 | 0.668 | 0.635 |
| NaiveBayes | 0.614(0.584 - 0.643) | 0.454 | 0.709 | 0.453 |
| SVM | 0.702(0.663 - 0.742) | 0.977 | 0.852 | 0.977 |
| RandomForest | 0.902(0.879 - 0.923) | 0.990 | 0.755 | 0.991 |
| XGBoost | 0.711(0.682 - 0.738) | 0.708 | 0.601 | 0.709 |
| LightGBM | 0.722(0.693 - 0.751) | 0.774 | 0.571 | 0.775 |
| GradientBoosting | 0.692(0.665 - 0.719) | 0.714 | 0.580 | 0.715 |
| AdaBoost | 0.697(0.670 - 0.723) | 0.717 | 0.555 | 0.718 |
| MLP | 0.701(0.674 - 0.728) | 0.679 | 0.612 | 0.680 |
| 3 years |  |  |  |  |
| LR | 0.700(0.682 - 0.717) | 0.655 | 0.626 | 0.655 |
| NaiveBayes | 0.612(0.592 - 0.631) | 0.602 | 0.563 | 0.603 |
| SVM | 0.590(0.558 - 0.621) | 0.982 | 0.879 | 0.983 |
| RandomForest | 0.582(0.552 - 0.608) | 0.980 | 0.827 | 0.982 |
| XGBoost | 0.722(0.704 - 0.739) | 0.618 | 0.691 | 0.617 |
| LightGBM | 0.706(0.688 - 0.724) | 0.778 | 0.503 | 0.781 |
| GradientBoosting | 0.667(0.648 - 0.685) | 0.796 | 0.448 | 0.800 |
| AdaBoost | 0.687(0.670 - 0.704) | 0.608 | 0.669 | 0.608 |
| MLP | 0.706(0.688 - 0.723) | 0.674 | 0.614 | 0.675 |
| 5 years |  |  |  |  |
| LR | 0.717(0.706 - 0.727) | 0.783 | 0.530 | 0.790 |
| NaiveBayes | 0.640(0.628 - 0.651) | 0.719 | 0.476 | 0.727 |
| SVM | 0.742 (0.724 - 0.760) | 0.959 | 0.855 | 0.962 |
| RandomForest | 0.831 (0.821 - 0.839) | 0.975 | 0.762 | 0.981 |
| XGBoost | 0.740(0.730 - 0.750) | 0.711 | 0.637 | 0.713 |
| LightGBM | 0.724(0.713 - 0.734) | 0.794 | 0.533 | 0.802 |
| GradientBoosting | 0.700(0.689 - 0.710) | 0.750 | 0.534 | 0.756 |
| AdaBoost | 0.690(0.679 - 0.700) | 0.632 | 0.652 | 0.631 |
| MLP | 0.742(0.732 - 0.752) | 0.718 | 0.636 | 0.721 |
| 12 years |  |  |  |  |
| LR | 0.748(0.743 - 0.752) | 0.694 | 0.669 | 0.698 |
| NaiveBayes | 0.717(0.711 - 0.721) | 0.668 | 0.643 | 0.672 |
| SVM | 0.741(0.733 - 0.749) | 0.850 | 0.784 | 0.861 |
| RandomForest | 0.741(0.734 - 0.750) | 0.938 | 0.853 | 0.952 |
| XGBoost | 0.766(0.761 - 0.770) | 0.712 | 0.677 | 0.718 |
| LightGBM | 0.747(0.742 - 0.752) | 0.705 | 0.657 | 0.713 |
| GradientBoosting | 0.736(0.731 - 0.741) | 0.691 | 0.656 | 0.697 |
| AdaBoost | 0.735(0.730 - 0.739) | 0.672 | 0.681 | 0.671 |
| MLP | 0.781 (0.778 - 0.786) | 0.748 | 0.766 | 0.745 |

**Table S8.** Performance comparison of mainstream ML algorithm applied for feature extractor across validation sets for predicting COPD-cause mortality.

| Model | AUC (95% CI) | Accuracy | Sensitivity | Specificity |
| --- | --- | --- | --- | --- |
| 1 year |  |  |  |  |
| LR | 0.883(0.775 - 0.991) | 0.892 | 0.600 | 0.892 |
| NaiveBayes | 0.773(0.603 - 0.942) | 0.512 | 0.800 | 0.512 |
| SVM | 0.761(0.574 - 0.947) | 0.521 | 0.800 | 0.521 |
| RandomForest | 0.693(0.453 - 0.932) | 0.845 | 0.425 | 0.873 |
| XGBoost | 0.583(0.379 - 0.787) | 0.782 | 0.425 | 0.812 |
| LightGBM | 0.506(0.204 - 0.808) | 0.798 | 0.438 | 0.825 |
| GradientBoosting | 0.581(0.382 - 0.780) | 0.792 | 0.428 | 0.818 |
| AdaBoost | 0.842(0.784 - 0.892) | 0.899 | 0.800 | 0.899 |
| MLP | 0.758(0.589 - 0.926) | 0.548 | 0.800 | 0.548 |
| 2 years |  |  |  |  |
| LR | 0.922(0.865 - 0.979) | 0.881 | 0.750 | 0.881 |
| NaiveBayes | 0.816(0.694 - 0.937) | 0.522 | 0.875 | 0.522 |
| SVM | 0.846(0.711 - 0.980) | 0.860 | 0.625 | 0.860 |
| RandomForest | 0.681(0.502 - 0.860) | 0.835 | 0.585 | 0.868 |
| XGBoost | 0.671(0.486 - 0.855) | 0.978 | 0.250 | 0.978 |
| LightGBM | 0.732(0.543 - 0.921) | 0.964 | 0.375 | 0.964 |
| GradientBoosting | 0.667(0.486 - 0.847) | 0.967 | 0.250 | 0.967 |
| AdaBoost | 0.947(0.914 - 0.979) | 0.890 | 0.875 | 0.890 |
| MLP | 0.715(0.521 - 0.909) | 0.604 | 0.750 | 0.604 |
| 3 years |  |  |  |  |
| LR | 0.864(0.788 - 0.940) | 0.866 | 0.650 | 0.866 |
| NaiveBayes | 0.753(0.657 - 0.848) | 0.612 | 0.750 | 0.612 |
| SVM | 0.813(0.605 - 0.960) | 0.929 | 0.800 | 0.929 |
| RandomForest | 0.795(0.684 - 0.905) | 0.998 | 0.350 | 0.998 |
| XGBoost | 0.630(0.524 - 0.734) | 0.976 | 0.250 | 0.976 |
| LightGBM | 0.656(0.546 - 0.766) | 0.958 | 0.300 | 0.959 |
| GradientBoosting | 0.603(0.506 - 0.700) | 0.963 | 0.200 | 0.964 |
| AdaBoost | 0.925(0.889 - 0.960) | 0.794 | 0.900 | 0.794 |
| MLP | 0.648(0.519 - 0.776) | 0.738 | 0.450 | 0.738 |
| 5 years |  |  |  |  |
| LR | 0.875(0.818 - 0.923) | 0.865 | 0.796 | 0.865 |
| NaiveBayes | 0.736(0.684 - 0.787) | 0.686 | 0.670 | 0.686 |
| SVM | 0.732(0.652 - 0.809) | 0.977 | 0.883 | 0.977 |
| RandomForest | 0.875(0.810 - 0.931) | 0.995 | 0.631 | 0.996 |
| XGBoost | 0.855(0.811 - 0.898) | 0.777 | 0.816 | 0.777 |
| LightGBM | 0.839(0.792 - 0.885) | 0.894 | 0.680 | 0.894 |
| GradientBoosting | 0.728(0.677 - 0.778) | 0.912 | 0.505 | 0.913 |
| AdaBoost | 0.890(0.859 - 0.921) | 0.812 | 0.835 | 0.812 |
| MLP | 0.863(0.831 - 0.893) | 0.848 | 0.689 | 0.848 |
| 12 years |  |  |  |  |
| LR | 0.818(0.800 - 0.835) | 0.720 | 0.773 | 0.720 |
| NaiveBayes | 0.660(0.636 - 0.682) | 0.703 | 0.541 | 0.704 |
| SVM | 0.977(0.970 - 0.983) | 0.975 | 0.887 | 0.976 |
| RandomForest | 0.969(0.961 - 0.977) | 0.984 | 0.814 | 0.986 |
| XGBoost | 0.819(0.800 - 0.838) | 0.867 | 0.659 | 0.869 |
| LightGBM | 0.814(0.795 - 0.833) | 0.872 | 0.626 | 0.874 |
| GradientBoosting | 0.761(0.741 - 0.779) | 0.683 | 0.726 | 0.683 |
| AdaBoost | 0.786(0.769 - 0.802) | 0.712 | 0.720 | 0.712 |
| MLP | 0.835(0.819 - 0.850) | 0.773 | 0.739 | 0.773 |

**Table S9.** Performance comparison of mainstream ML algorithm applied for feature extractor across validation sets for predicting lung cancer -cause mortality.

| Model | AUC (95% CI) | Accuracy | Sensitivity | Specificity |
| --- | --- | --- | --- | --- |
| 1 year |  |  |  |  |
| LR | 0.926(0.826 - 0.999) | 0.910 | 0.875 | 0.910 |
| NaiveBayes | 0.831(0.716 - 0.945) | 0.853 | 0.687 | 0.853 |
| SVM | 0.891(0.797 - 0.984) | 0.878 | 0.750 | 0.878 |
| RandomForest | 0.708(0.459 - 0.934) | 0.998 | 0.562 | 0.998 |
| XGBoost | 0.606(0.494 - 0.717) | 0.969 | 0.187 | 0.969 |
| LightGBM | 0.602(0.492 - 0.712) | 0.962 | 0.187 | 0.963 |
| GradientBoosting | 0.606(0.495 - 0.716) | 0.967 | 0.187 | 0.967 |
| AdaBoost | 0.973(0.963 - 0.983) | 0.944 | 0.875 | 0.944 |
| MLP | 0.919(0.861 - 0.977) | 0.909 | 0.750 | 0.910 |
| 2 years |  |  |  |  |
| LR | 0.866(0.827 - 0.903) | 0.663 | 0.921 | 0.663 |
| NaiveBayes | 0.866(0.827 - 0.903) | 0.663 | 0.921 | 0.663 |
| SVM | 0.541(0.466 - 0.615) | 0.979 | 0.270 | 0.979 |
| RandomForest | 0.636(0.588 - 0.683) | 0.978 | 0.292 | 0.979 |
| XGBoost | 0.769(0.715 - 0.822) | 0.881 | 0.629 | 0.881 |
| LightGBM | 0.863(0.825 - 0.899) | 0.798 | 0.787 | 0.798 |
| GradientBoosting | 0.717(0.663 - 0.770) | 0.942 | 0.483 | 0.943 |
| AdaBoost | 0.854(0.818 - 0.890) | 0.605 | 0.933 | 0.605 |
| MLP | 0.866(0.827 - 0.903) | 0.663 | 0.921 | 0.663 |
| 3 years |  |  |  |  |
| LR | 0.878(0.855 - 0.900) | 0.779 | 0.814 | 0.779 |
| NaiveBayes | 0.878(0.855 - 0.900) | 0.779 | 0.814 | 0.779 |
| SVM | 0.485(0.429 - 0.541) | 0.976 | 0.300 | 0.978 |
| RandomForest | 0.643(0.611 - 0.674) | 0.973 | 0.310 | 0.975 |
| XGBoost | 0.862(0.837 - 0.886) | 0.881 | 0.671 | 0.882 |
| LightGBM | 0.868(0.843 - 0.892) | 0.806 | 0.781 | 0.806 |
| GradientBoosting | 0.830(0.798 - 0.860) | 0.800 | 0.786 | 0.800 |
| AdaBoost | 0.867(0.843 - 0.890) | 0.800 | 0.786 | 0.800 |
| MLP | 0.878(0.855 - 0.900) | 0.779 | 0.814 | 0.779 |
| 5 years |  |  |  |  |
| LR | 0.919(0.909 - 0.929) | 0.846 | 0.829 | 0.846 |
| NaiveBayes | 0.840(0.822 - 0.857) | 0.800 | 0.729 | 0.800 |
| SVM | 0.806(0.773 - 0.836) | 0.972 | 0.961 | 0.973 |
| RandomForest | 0.498(0.456 - 0.534) | 0.909 | 0.898 | 0.909 |
| XGBoost | 0.912(0.900 - 0.923) | 0.849 | 0.808 | 0.850 |
| LightGBM | 0.909(0.897 - 0.921) | 0.828 | 0.821 | 0.828 |
| GradientBoosting | 0.874(0.861 - 0.886) | 0.817 | 0.779 | 0.818 |
| AdaBoost | 0.890(0.878 - 0.900) | 0.797 | 0.835 | 0.797 |
| MLP | 0.924(0.914 - 0.933) | 0.875 | 0.811 | 0.875 |
| 12 years |  |  |  |  |
| LR | 0.847(0.838 - 0.855) | 0.761 | 0.789 | 0.761 |
| NaiveBayes | 0.741(0.729 - 0.752) | 0.743 | 0.620 | 0.746 |
| SVM | 0.916(0.909 - 0.922) | 0.979 | 0.958 | 0.980 |
| RandomForest | 0.469(0.447 - 0.492) | 0.910 | 0.951 | 0.909 |
| XGBoost | 0.870(0.862 - 0.877) | 0.726 | 0.862 | 0.722 |
| LightGBM | 0.858(0.849 - 0.866) | 0.776 | 0.800 | 0.776 |
| GradientBoosting | 0.838(0.830 - 0.846) | 0.734 | 0.819 | 0.732 |
| AdaBoost | 0.835(0.827 - 0.843) | 0.743 | 0.774 | 0.742 |
| MLP | 0.811(0.797 - 0.826) | 0.971 | 0.964 | 0.971 |

**Table S10.** Performance comparison of mainstream ML algorithm applied for feature extractor across validation sets for predicting cardiac cause mortality.

| Model | AUC (95% CI) | Accuracy | Sensitivity | Specificity |
| --- | --- | --- | --- | --- |
| 1 year |  |  |  |  |
| LR | 0.570(0.451 - 0.688) | 0.263 | 0.840 | 0.263 |
| NaiveBayes | 0.652(0.545 - 0.757) | 0.637 | 0.680 | 0.637 |
| SVM | 0.925(0.855 - 0.995) | 0.900 | 0.840 | 0.900 |
| RandomForest | 0.836(0.741 - 0.929) | 0.998 | 0.440 | 0.998 |
| XGBoost | 0.520(0.464 - 0.575) | 0.982 | 0.040 | 0.983 |
| LightGBM | 0.484(0.445 - 0.522) | 0.018 | 0.960 | 0.018 |
| GradientBoosting | 0.500(0.460 - 0.538) | 0.983 | 0.000 | 0.983 |
| AdaBoost | 0.757(0.702 - 0.811) | 0.684 | 0.760 | 0.684 |
| MLP | 0.616(0.504 - 0.727) | 0.675 | 0.560 | 0.675 |
| 2 years |  |  |  |  |
| LR | 0.653(0.603 - 0.702) | 0.549 | 0.667 | 0.549 |
| NaiveBayes | 0.582(0.533 - 0.630) | 0.427 | 0.759 | 0.427 |
| SVM | 0.637(0.561 - 0.714) | 0.927 | 0.926 | 0.927 |
| RandomForest | 0.892(0.853 - 0.930) | 0.996 | 0.519 | 0.997 |
| XGBoost | 0.662(0.613 - 0.711) | 0.950 | 0.176 | 0.951 |
| LightGBM | 0.525(0.496 - 0.552) | 0.972 | 0.056 | 0.973 |
| GradientBoosting | 0.528(0.500 - 0.555) | 0.963 | 0.083 | 0.964 |
| AdaBoost | 0.670(0.622 - 0.717) | 0.566 | 0.704 | 0.565 |
| MLP | 0.626(0.579 - 0.673) | 0.246 | 0.954 | 0.245 |
| 3 years |  |  |  |  |
| LR | 0.661(0.629 - 0.692) | 0.674 | 0.575 | 0.674 |
| NaiveBayes | 0.595(0.561 - 0.628) | 0.452 | 0.697 | 0.451 |
| SVM | 0.750(0.707 - 0.793) | 0.981 | 0.866 | 0.982 |
| RandomForest | 0.616(0.560 - 0.673) | 0.993 | 0.674 | 0.994 |
| XGBoost | 0.677(0.645 - 0.709) | 0.627 | 0.613 | 0.627 |
| LightGBM | 0.641(0.607 - 0.675) | 0.624 | 0.594 | 0.624 |
| GradientBoosting | 0.527(0.508 - 0.544) | 0.964 | 0.080 | 0.966 |
| AdaBoost | 0.659(0.628 - 0.688) | 0.642 | 0.602 | 0.642 |
| MLP | 0.682(0.651 - 0.712) | 0.689 | 0.571 | 0.689 |
| 5 years |  |  |  |  |
| LR | 0.680(0.659 - 0.700) | 0.561 | 0.707 | 0.560 |
| NaiveBayes | 0.579(0.556 - 0.601) | 0.728 | 0.391 | 0.730 |
| SVM | 0.778(0.746 - 0.811) | 0.980 | 0.854 | 0.981 |
| RandomForest | 0.504(0.464 - 0.547) | 0.989 | 0.788 | 0.990 |
| XGBoost | 0.703(0.681 - 0.723) | 0.581 | 0.722 | 0.580 |
| LightGBM | 0.687(0.663 - 0.709) | 0.709 | 0.580 | 0.710 |
| GradientBoosting | 0.598(0.578 - 0.618) | 0.957 | 0.099 | 0.963 |
| AdaBoost | 0.652(0.632 - 0.672) | 0.567 | 0.662 | 0.567 |
| MLP | 0.708(0.688 - 0.727) | 0.672 | 0.643 | 0.672 |
| 12 years |  |  |  |  |
| LR | 0.712(0.702 - 0.720) | 0.658 | 0.662 | 0.657 |
| NaiveBayes | 0.599(0.589 - 0.609) | 0.548 | 0.607 | 0.546 |
| SVM | 0.760(0.744 - 0.774) | 0.955 | 0.840 | 0.960 |
| RandomForest | 0.760(0.746 - 0.773) | 0.971 | 0.796 | 0.977 |
| XGBoost | 0.754(0.745 - 0.762) | 0.706 | 0.678 | 0.708 |
| LightGBM | 0.736(0.726 - 0.744) | 0.683 | 0.676 | 0.683 |
| GradientBoosting | 0.691(0.681 - 0.700) | 0.683 | 0.613 | 0.686 |
| AdaBoost | 0.703(0.694 - 0.712) | 0.608 | 0.718 | 0.604 |
| MLP | 0.777(0.769 - 0.785) | 0.672 | 0.754 | 0.669 |

**Table S11.** Comprehensive metadata for CXR imaging parameters

| Parameters | CXRs |
| --- | --- |
| Projection Direction | Posterior-anterior |
| Tube Voltage (kVp) | 105-110 |
| Exposure (mAs) | 8-12 |
| Exposure Time (s) | 3 |
| Source-to-Image Distance (SID, cm) | 180 |
| Standing height (cm) | 150 |
| X-ray system | Kodak DR3500 |
| Pixel Spacing (mm/pixel) | 0.143 (commonly for 14x14 inch detector) |
| Bits Allocated | 16 |
| Bits Stored | 12 |
| Image Contrast | High |
| Signal-to-Noise Ratio (SNR) | ≥ 40 |


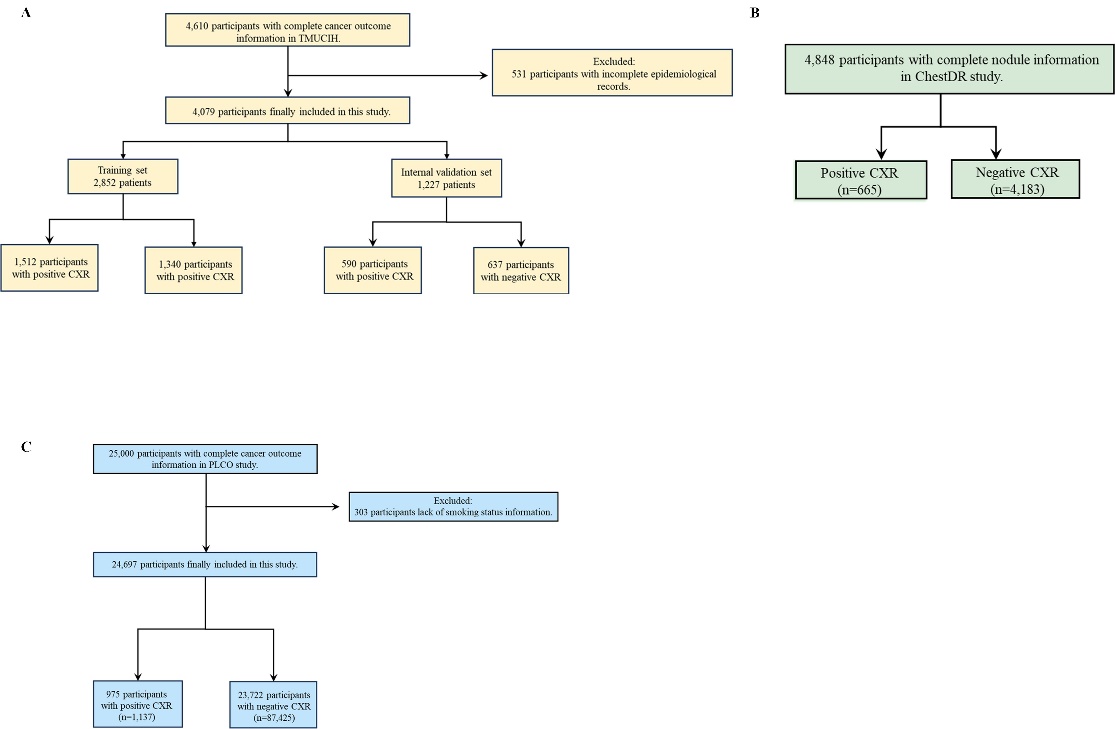


**Figure S1.** Flow chart of patient inclusion and exclusion in (A) TLCID, (B) ChestDR and (C) PLCO. CXR: chest X-ray; TMUCIH, Tianjin Medical University Cancer Institute and Hospital; PLCO: The Prostate, Lung, Colorectal and Ovarian Cancer Screening Trial; TLCID: Tianjin Lung Cancer Imaging Dataset.


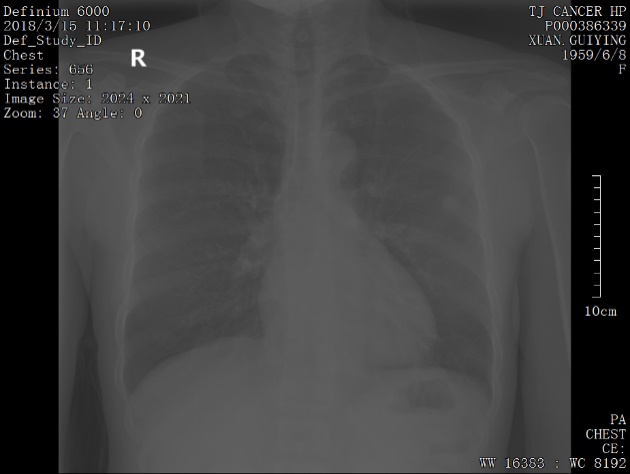
(A)
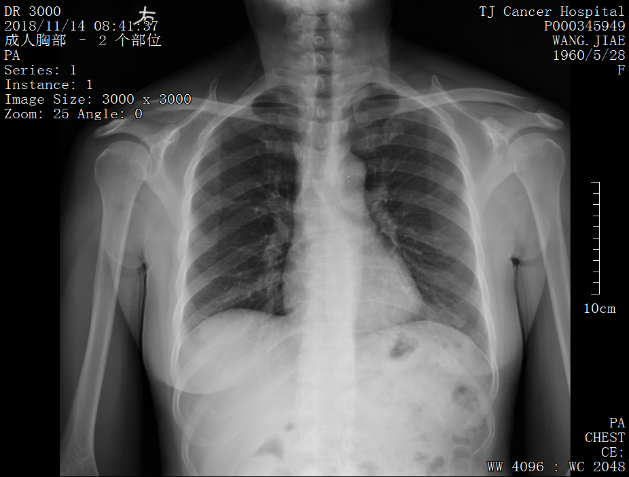
 (B) (C)


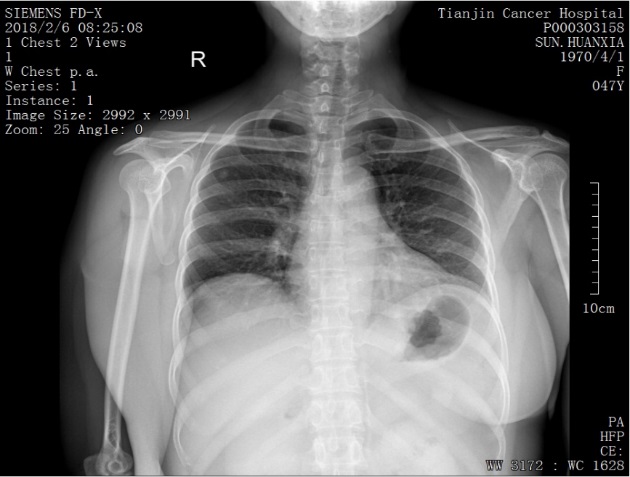


**Figure S2.** Representative examples of chest X-rays with pulmonary nodules of different diameters. **(A)** Pulmonary nodule with a diameter of less than 5 mm.**(B)** Pulmonary nodule with a diameter between 5 mm and 10 mm.**(C)** Pulmonary nodule with a diameter greater than 10 mm.
